# *N*-Acetylaspartyl-Glutamate Metabolism in the Cingulated Cortices as a Biomarker of the Etiology in ASD: A ^1^H-MRS Model

**DOI:** 10.3390/molecules26030675

**Published:** 2021-01-28

**Authors:** Carmen Jiménez-Espinoza, Francisco Marcano Serrano, José Luis González-Mora

**Affiliations:** 1 Laboratory Neurochemistry & Neuroimages, Department of Basic Medical Sciences, Faculty of Health Sciences, Physiology Section, University of La Laguna, 38200 Tenerife, Spain; fmarcano@ull.es (F.M.S.); jlgonzal@ull.edu.es (J.L.G.-M.); 2 Magnetic Resonance Service for Biomedical Research (SRMIB), IMETISA, Canary University Hospital, 38320 Tenerife, Spain

**Keywords:** autism spectrum disorder, brain metabolism, *N*-Acetyl-aspartyl-glutamate, biomarkers, resonance magnetic spectroscopy, cingulated cortices

## Abstract

As brain functional resonance magnetic studies show an aberrant trajectory of neurodevelopment, it is reasonable to predict that the degree of neurochemical abnormalities indexed by magnetic resonance spectroscopy (^1^H-MRS) might also change according to the developmental stages and brain regions in autism spectrum disorders (ASDs). Since specific *N*-Acetyl-aspartate (NAA) changes in children’s metabolism have been found in the anterior cingulate cortex (ACC) but not in the posterior cingulate cortex (PCC), we analyzed whether the metabolites of ASD youths change between the cingulate cortices using ^1^H-MRS. l-glutamate (Glu) and l-Acetyl-aspartate (NAA) are products from the *N*-Acetyl-aspartyl-glutamate (NAAG) metabolism in a reaction that requires the participation of neurons, oligodendrocytes, and astrocytes. This altered tri-cellular metabolism has been described in several neurological diseases, but not in ASD. Compared to the typical development (TD) group, the ASD group had an abnormal pattern of metabolites in the ACC, with a significant increase of glutamate (12.10 ± 3.92 mM; *p* = 0.02); additionally, *N*-Acetyl-aspartyl-glutamate significantly decreased (0.41 ± 0.27 mM; *p* = 0.02) within ASD metabolism abnormalities in the ACC, which may allow the development of new therapeutic possibilities.

## 1. Introduction

Autism spectrum disorders (ASDs) are neurodevelopment disorders with early life onset and a variable developmental trajectory, primarily affecting high-order integration processes, such as complex social interactions, associative thinking, and appropriate emotional reactions. Social and self-referential cognitive processes have been linked with a pair of cortical midline brain regions, the ventromedial prefrontal cortex (VMPFC) and the posterior cingulated cortex (PCC), which serve as hubs of the default mode network (DMN) [1]. Structurally, the PCC is part of the cingulated gyros, which is a major part of the anatomical limbic system in the brain and, according to classic accounts, is involved in emotions [2].

Even so, there is no clear consensus about the function and importance of the PCC in health and disease [3]. However, evidence suggests that the PCC is highly heterogeneous and may play a direct role in regulating the focus of the attention linked to the attention network system, which is of the same category as the motor and sensory systems in healthy subjects. In support of this, post-mortem studies have provided evidence for cytoarchitectonic abnormalities within the PCC of patients with ASD, compatible with an abnormality in local circuit inhibition [4], as well as with functional abnormalities—specifically, functional responses and reductions in functional connectivity.

However, it is known that the DMN may be largely intact but under-utilized, perhaps because of a dysfunction in the control systems that regulate its use in ASD [5]. Indeed, a reduction in the connectivity between the PCC and the medial prefrontal cortex in a mixed group of children and young adolescents with this disorder has been reported [6]. Moreover, abnormalities in cingulated responses during interpersonal interaction correlate with the severity of patients’ autistic symptoms [7], with a correlation between overall social functioning and failure of task-dependent deactivation in the PCC [8].

Recently, a new network linked to the pathophysiology of ASD has emerged, called the salience network (SAN), and its dysfunction may be part of the underlying neurobiology of autism [9]. It is associated with the anterior insula and the anterior cingulated cortex (ACC), the two main nodes of the SAN, linked to the behaviors affected in autism, ranging from social perception to cognitive control [10]. Furthermore, it has been demonstrated that this network is involved in interoceptive and affective processes, as well as the identification of relevant internal and external personal stimuli that lead to certain behavior [11,12].

In support of this, magnetic resonance spectroscopy has many advantages for developmental psychiatry because it provides important chemical information on metabolites; furthermore, it can be useful to explaining the aberrant trajectories of brain development in ASD that have not yet been elucidated. Considering studies of brain functioning showing an aberrant trajectory of neurodevelopment, it is reasonable to predict that the degree of neurochemical abnormalities indexed by ^1^H-MRS may also change according to the developmental stages and brain regions in ASD [13]. In this sense, our previous work showed evidence of abnormalities in the absolute concentrations of neurometabolites related to the ACC and PCC [14]. Contrarily, in healthy subjects, it has been shown that although each ACC and PCC region has different functions, they maintain the metabolic balance.

Taking into account all of the presented evidence, our goal was to study the neurometabolic link between the anterior and posterior cingulated cortices using the detected principal metabolites by ^1^H-MRS, as markers of changes in the neuron density, glial density, cell membrane processes, and energetic metabolism of brain tissue in subjects with ASD. One of our principal findings was the imbalance of the absolute concentration of *N*-Acetyl-aspartyl-glutamate (NAAG) between the ACC and the PCC, parallel with a significantly increased glutamate (Glu) concentration in the ACC, which correlated with the severity level of the symptoms of ASD. Moreover, other authors have shown an increasing level of *N*-Acetyl-aspartate (NAA) in the prefrontal area, alongside increased neuronal attenuation or metabolic abnormality and/or abnormal synaptic pruning in adults with Asperger’s syndrome [15], confirming the altered metabolism of NAAG reported in this work.

We also considered it important to highlight that many of the causes of the local elevation of NAA result from multiple metabolic mechanisms, including faster astrocyte membrane decomposition of NAAG into Glu and NAA [16], slower oligodendrocyte membrane degradation of NAA into acetate and aspartate [17,18], slower intraneuronal synthesis of NAAG out of NAA and Glu [19], and/or faster intraneuronal NAA synthesis out of aspartate and acetyl-CoA [20]. Furthermore, such an excess of NAA may also reflect increased mitochondrial metabolism [21]. In contrast, the Glu is the major excitatory neurotransmitter in the brain, which plays a major role in brain development, causing perturbations in neuronal migration, neuronal differentiation, axon genesis, and neuronal survival [22]. Our findings suggest that the abnormal physiology in ASD includes disturbances in the Glu and/or NAA neurometabolism and the signal into the ACC and the PCC, as one locus of abnormality into the cingulated gyrus, which reflects the relevance of cellular neurochemistry in the pathological progression and severity of ASD [23].

### 1.1. N-Acetylaspartyl-Glutamate Synthesis

NAAG is the most abundant neuropeptide in the central nervous system of mammals, present in high micromolar to low millimolar concentrations [17], and the highest concentrations are found in the spinal cord and brain stem [24]. This peptide, called a neurotransmitter, was recently shown to be synthesized by a neuron-specific synthetase, catalyzing the ATP-dependent condensation of NAA and Glu [19], both of which are present in the brain. On the one hand, NAAG is synthesized from NAA and Glu by a NAAG synthetase, forming a Glu pool that cannot be further metabolized, while on the other hand, NAAG is hydrolyzed by NAAG peptidase, thereby releasing Glu, which activates the type 3 metabotropic glutamate receptor (mGluR3) receptor [25].

### 1.2. Properties of NAAG

Imbalances in the concentration of NAAG affect long-term potentiation and depression in the hippocampus [26]. In addition, NAAG synthesis modulates the functioning of various synapses—such as γ-aminobutyric acid, acetylcholine, and glutamate—in the cortex, amygdala, hippocampus, striatum, brain stem, and spinal cord, which is why this peptide is related to attention/concentration and memory (cognition) [27].

Another property of NAAG is its activation of presynaptic metabotropic receptors, which reduces glutamate release; by exciting the receptor located in the fibrillar astrocytes, it releases a protective factor from the synapses called β-growth factor. In contrast, it postsynaptically desensitizes and blocks *N*-methyl-d-aspartate [28]. Although the effects of NAAG appear to be mediated by its agonistic binding to the mGluR3, a previous study has suggested that NAAG may not be an agonist of mGluR3 [29]. Furthermore, NAAG is also considered essential for the progress of a neuronal interaction called synaptic plasticity because its metabolism allows it to be sustained and efficient; to achieve this, it is essential to reconcile the presence of glial cells [18,30]. In contrast, altered NAAG metabolism has been described in some neurological conditions such as schizophrenia (one of the diseases linked with autism diagnostics at the beginning), amyotrophic lateral sclerosis, and traumatic brain injury, but not ASD [31,32].

Taking into account previous findings, the potential relationship of NAAG metabolism aberrations and the ACC and PCC still remains unexplored in ASD. On the basis of these considerations, we designed the present study to determine if it is possible to measure the NAAG levels in specific brain areas, allowing therapeutic diagnostics. In was assumed that accurate characterization of neurometabolites in the brain in ASD is the first step toward developing new biomarkers for autism diagnostics, which suggests a new and rising therapeutic avenue. Despite the previous studies [13] that have not reported an increase or decrease in the signaling of metabolites in the PCC region, the results herein demonstrate the first direct evidence of the relationship between abnormal metabolic activity and PCC dysfunction in ASD.

## 2. Results

There were no significant group differences in terms of demographic variables (i.e., sex and age). However, there were group differences in the domains of the Autism-Spectrum Quotient (AQ) test (i.e., attention switching, attention to detail, communication, and imagination). As expected, there was a significant group difference in the social skills AQ domain (*p* = 0.04). In addition, there were significant group differences in physical symptoms (epilepsy: *p* < 0.0015; gastrointestinal disorders: *p* < 0.0001; muscular hypotonic: *p* < 0.0001; familiar hypothyroidism: *p* < 0.0001). Conventional MR imaging of all subjects did not show any abnormalities (see Table 1).

### 2.1. H-MRS (Magnetic Resonance Spectroscopy)

#### 2.1.1. ^1^H-MRS in the Cingulated Cortices in ASD

**Figure 1:**
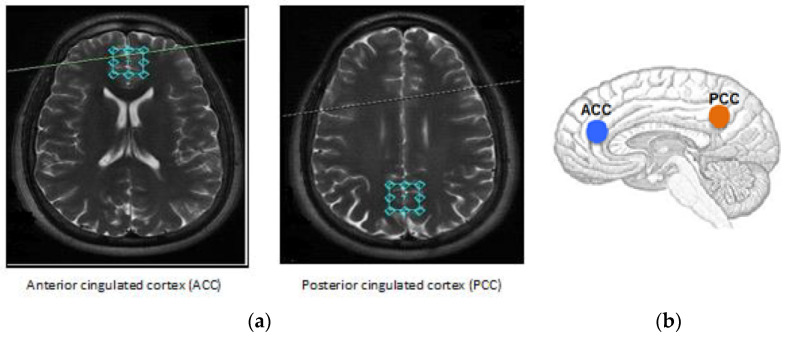
Locations of the volume studied in the (**a**) anterior and (**b**) posterior cingulated cortices. The single voxel acquisition used a spin-echo sequence recorded within the following parameters: Echo time (TE) = 23 ms, repetition time (TR) = 1070 ms, 2 NEX, flip angle = 90°, and 256 acquisitions with the point-resolved spectroscopy (PRESS) technique. During data acquisition, the same experienced neuroradiologist, blind to the clinical data, placed the voxels (20 × 20 × 20 mm^3^) at the ACC and PCC.

During data acquisition, the same experienced neuroradiologist, blind to the clinical data, placed the voxels (2 × 2 × 2 cm^3^) at the ACC and PCC (see Figure 1). The ASD group had an abnormal pattern of metabolites in the ACC with a significant increase in glutamate (12.10 ± 3.92 mM; *p* = 0.02), as well as a significant decrease in *N*-acetylaspartyl-glutamate (0.41 ± 0.27 mM; *p* = 0.02), compared to the typical development (TD) group (see Table 2).

Moreover, important differences in NAA/creatine (Cr) (t = 0.02) and myoinositol (mI)/Cr (t = 0.04) ratios were calculated in relation to creatine, in contrast to the significant difference in NAA/ choline (Cho) (t = 0.01) in the PCC (see Table 3). On the contrary, there was a significant difference in NAA/Cho both in ASD (*p* = 0.0001) and in TD (*p* < 0.0001) in the ACC (see Table 4) when compared to the NAA ratios (NAA/Cr, NAA/mI, and NAA/Cho) between the ACC and PCC in each group, considering NAA as a marker of mitochondrial metabolism.

#### 2.1.2. ^1^H-MRS and the Subgroups of Autism Severity (AQ1, AQ2, AQ3, and AQ4)

**Figure 2:**
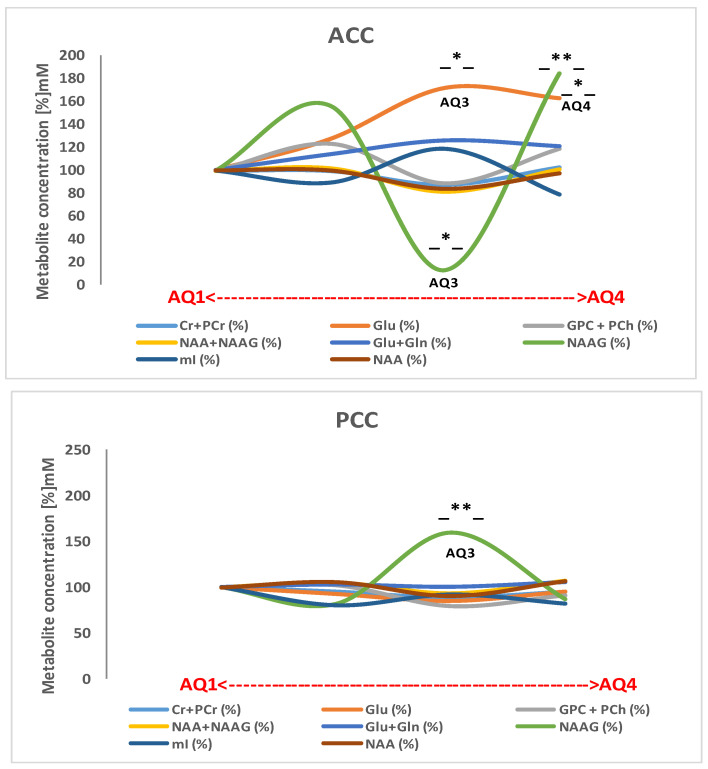
Absolute concentration (mM) patterns of the different metabolites detected in the ACC and PCC in all AQ indexes (AQ1 = 28.3%, *n* = 17; AQ2 = 43.3%, *n* = 26; AQ3 = 10.0%, *n* = 9; AQ4 = 18.3%, *n* = 13) as a marker of ASD severity. The X-axis trajectories within the AQ range are represented in the normalized graph. * *p* < 0.05. -*-, -**-; significant at *p* < 0.05; 0.01; respectively.

To examine whether the absolute metabolic concentrations in the different subgroups with different levels of severity of autism are associated, four thresholds were used (AQ1, AQ2, AQ3, and AQ4) (see Table 5 and Figure 2).

**Figure 3:**
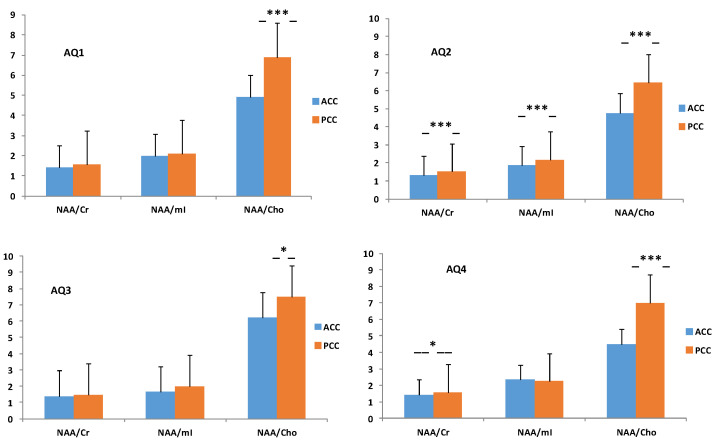
Differences in the means of the NAA/Cr, NAA/mI, and NAA/Cho ratios between the ACC and PCC in the AQ1 (28.3%; *n* = 17), AQ2 (43.3%; *n* = 26), AQ3 (10.0%; *n* = 9), and AQ4 (18.3%; *n* = 13) groups. The AQ1 group was taken as a reference or control. *p* < 0.05 considered significantly different. -*-, -***-, significant at *p* < 0.05, 0.001, respectively.

In addition, regarding the ratio of metabolites between the ACC and the PCC for each subgroup stratified empirically by [33], these categories, derived by the AQ test, index the severity of the symptoms of autism. There was a significant difference in the ratio proportions (see Table 6) between the ACC and the PCC in AQ1 (NAA/Cho: *p* = 0.0004), AQ2 (NAA/Cr: *p* = 0.0002; NAA/mI: *p* = 0.0003; NAA/Cho: *p* < 0.0001), AQ3 (NAA/Cho: *p* = 0.05), and AQ4 (NAA/Cr: *p* = 0.05; NAA/Cho: *p* < 0.0001) (see Figure 3).

### 2.2. Imbalance of N-Acetylaspartyl-Glutamate Metabolism in ASD

The metabolic pathway that this neuropeptide follows in nerve cells is divided into two conjugated processes, namely, catabolism and anabolism. The catabolic reaction releases energy, which, in turn, is used by the anabolic reaction to recompose the chemical bonds to form the protein. These processes are coupled, since one depends on the other. NAAG is released to the extracellular space synaptically, where it is inactivated by the two peptides located in the glial cell, called glutamate carboxypeptidase II and III (GCPII and GCPIII) [27], and reuptaken by the postsynaptic neuron through the NMDA receptor.

**Figure 4:**
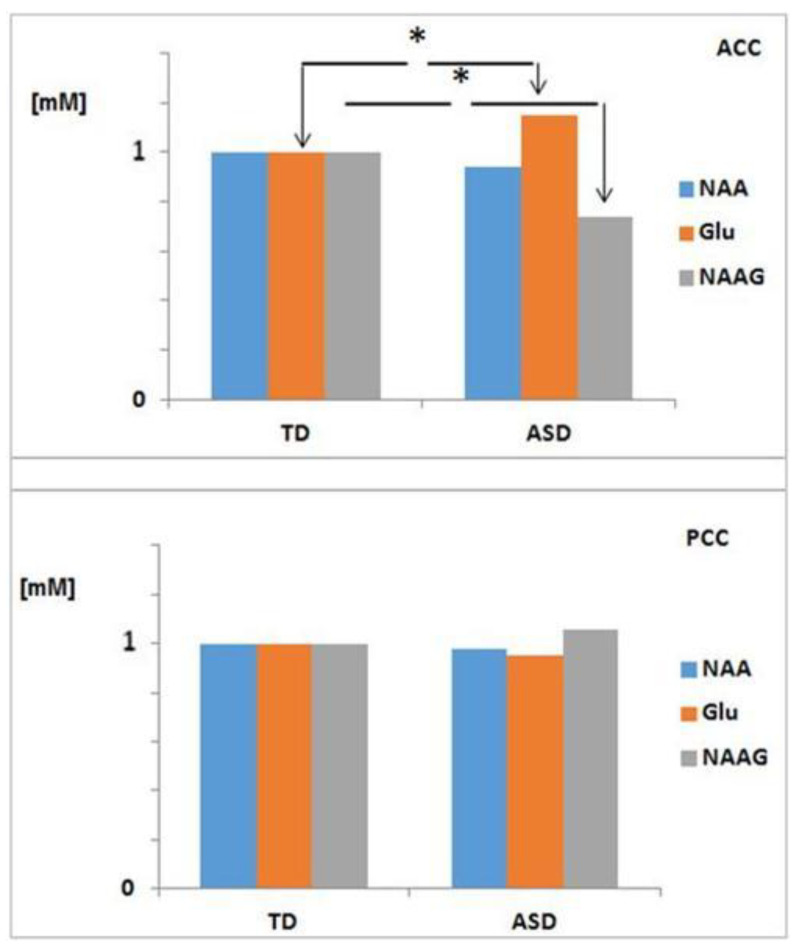
Differences in the means of the normalized absolute concentrations of NAA, NAAG, and Glu in ASD (*n* = 22) and TD (*n* = 43), in both the ACC and the PCC. * *p* < 0.05 considered significantly different. *p* < 0.05 considered significantly different. *, significant at *p* < 0.05,

**Figure 5:**
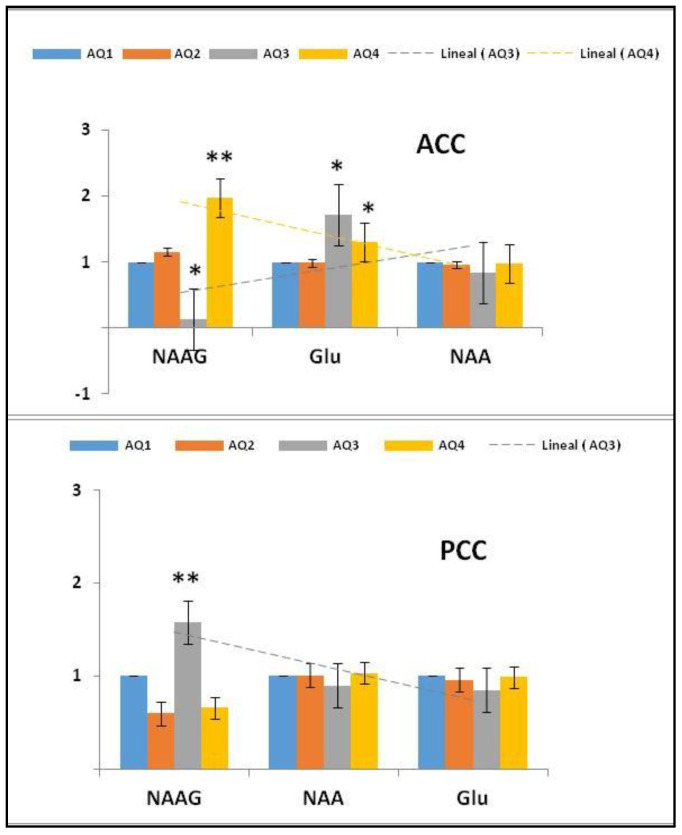
Mean absolute concentrations of NAA, NAAG, and Glu in NAAG metabolism in the ACC and PCC within each AQ group (AQ1 = 28.3%, *n* = 17; AQ2 = 43.3%, *n* = 26; AQ3 = 10.0%, *n* = 9; AQ4 = 18.3%, *n* = 13), with the AQ1 group taken as a reference or control and the AQ index as a marker of ASD severity. Standardized graph, *p* < 0.05 considered significantly different. *, **, significant at *p* < 0.05; 0.01; respectively.

Given the finding of a deregulation of NAAG metabolism in the cingulated cortices in the group with ASD (NAAG: * *p* = 0.02; Glu: * *p* = 0.02) in the ACC (see Table 2 and Figure 4), we considered the convenience of evaluating NAAG metabolism in the study population grouped by AQ index. In this sense, in the AQ3 and AQ4 groups, a significant increase in the concentration of glutamate was observed in the ACC (13.87 ± 2.13 and 13.14 ± 4.14 mM, respectively) as compared to AQ1 (healthy controls). Moreover, a significant decrease in NAAG (0.21 ± 0.46 mM) in the AQ3 group and a very significant increase (0.81 ± 1.39 MM) in AQ4 (see Figure 5) led us to suggest that the biosynthesis of NAAG in the AQ3 group follows the catabolic pathway, where the cellular toxicity produced by the accumulation of glutamate in the ACC could be the cause of the inherent deficits in the hypoconnectivity of the SAN and the DMN described in children with ASD [34]; Conversely, in the AQ4 group, it follows the anabolic pathway.

(1) $$\frac{\mathrm{NAA}\;+\;\mathrm{Glu}\;}{\begin{array}{c} \mathrm{Anabolic}\\ \mathrm{pathway} \end{array}}\to \mathrm{NAAG}\to \;\frac{\mathrm{NAA}\;+\;\mathrm{Glu}}{\begin{array}{c} \mathrm{Catabolic}\\ \mathrm{pathway} \end{array}}$$

**Figure 6:**
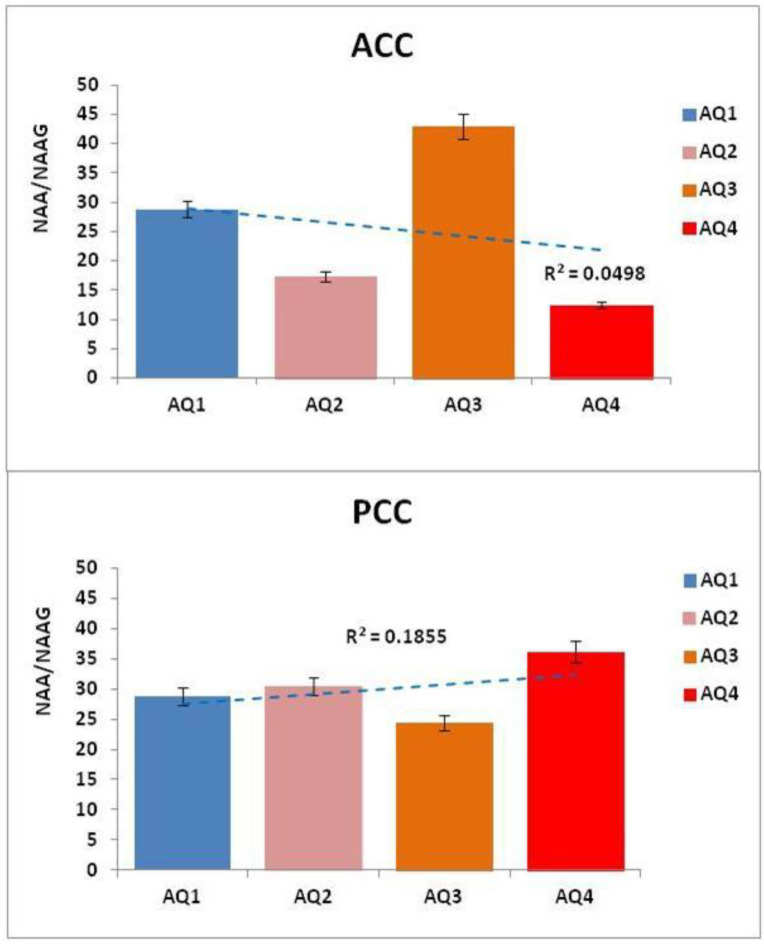
Mean of the NAA/NAAG ratios in the ACC and PCC within each AQ group (AQ1 = 28.3%, *n* = 17; AQ2 = 43.3%, *n* = 26; AQ3 = 10.0%, *n* = 9; AQ4 = 18.3%, *n* = 13), with the AQ1 group taken as a reference or control and the AQ index as a marker of ASD severity. *p* < 0.05 considered significantly different.

In contrast to the ACC, where the index AQ4 subjects showed a very significant increase in NAAG (88.37%), the findings in the PCC showed a significant increase in NAAG (17.14%) in AQ3 subjects with respect to AQ1. This suggests a relationship between the imbalance of NAAG in the PCC with the social and self-referential cognitive processes disrupted in ASD. On the contrary, “The structures, metabolism, and dynamics of NAA and NAAG are intertwined, as NAAG is synthesized in the normal brain from NAA at a constant rate, maintained at a constant NAA/NAAG ratio, both liberated to ECF at the same rate and also catabolized at the same rate in order to maintain their constant brain metabolite ratio” [35]. Considering this, we calculated the NAA/NAAG ratio in the ACC and PCC in all AQ indexes as marker of ASD severity. The results showed a clear imbalance of the NAA/NAAG ratio in AQ2, AQ3, and AQ4 with respect to AQ1, evidencing changes in all autism spectrum groups (see Table 7 and Figure 6).

#### *N*-Acetylaspartyl-Glutamate Biosynthesis: Kinetics Model In Vivo

The enzyme(s) synthesizing NAAG were not characterized in vitro despite; the high expression levels of NAAGS restricted to the brain has been strongly suggested to permit the identification of the gene that encodes NAAG synthetase [36].

Through the study of the kinetics and chemical dynamics of an enzyme that we can explain the details of its catalytic mechanism, its role in metabolism, how its activity in the cell is controlled, and how its activity can be inhibited by drugs, or powered by other types of molecules. The Michaelis–Menten kinetics is one of the best-known models of enzyme kinetics, that can be used to characterize a generic biochemical reaction.

Although, this model is used in a variety of biochemical situations other than enzyme–substrate interaction; including antigen–antibody binding, DNA–DNA hybridization, and protein–protein interaction [37].

Here, the Michaelis–Menten equation model, simply out, does not fit the data very well. Therefore, such results are unlikely to provide useful information. However, with the substrates inhibition model, a very significant decrease in the dissociation constant (Ki) for glutamate binding (Ki(Glu) = 1.04 mM; R^2^ = −14.72) in the ASD group was observed, compared to the control group (TD) (Ki(Glu) = 15.26 mM; R^2^ = −20.33), in the PCC (Table 8), showing the more tightly bound the enzyme, the higher the affinity between the enzyme and substrate (NAAGSGlu). In contrast, the Michaelis–Menten constant (km(Glu) = 4.68 × 10^−3^ mM, R^2^ = −14.72; km(NAA) = 2.92 × 10^−3^ mM, R^2^ = −23.95) was highest in ASD, suggesting a lower affinity of the enzyme for the substrate, which could be related to the great stability of the NAAGSGlu complex in the PCC compared to TD. On the contrary, a comparison between ASD and TD for NAA binding was not possible, as it did show the constants in the TD group, which indicates an increase in the affinity of the enzyme for the substrate, and also because it needs less substrate to bind 50% of the enzyme.

With respect to the ACC, we noticed that the Michaelis–Menten constant (km(Glu) = 5.9 × 10^6^ mM, R^2^ = −9.8; km(NAA) = 5.8 × 10^6^ mM, R^2^ = −27.05) was decreased in ASD compared to TD, suggesting increased NAAGSNAAGlu complex stability.

**Figure 7:**
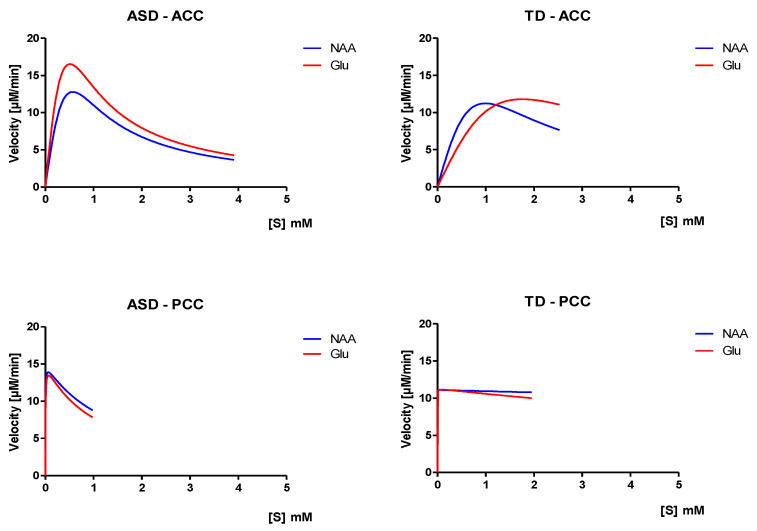
Diagram of the reaction rate and constant of Michaelis–Menten (Km) as a function of the substrate inhibition in *N*-Acetylaspartyl-glutamate enzyme kinetics. *p* < 0.05 considered significantly different. (ASD: *n* = 22; TD: *n* = 43).

Finally, V_max_ of the appearance of the product that depends on the slowest process of the reaction was significantly increased (V_max_(Glu) = 19.13 µM/min; R^2^ = −14.72) in subjects with ADS in the PCC (see Figure 7).

## 3. Discussion

Social and self-referential cognitive processes are linked to the PCC, which serves as a hub of the DMN. Even alterations in this structure can trigger pathological effects, evidence of which suggests that this region is highly heterogeneous and may play a direct role in regulating the focus of attention in healthy subjects. In support of this, post-mortem studies have provided evidence for cytoarchitectonic abnormalities within the PCC of patients with ASD [4], compatible with an abnormality in local circuit inhibition, as well as with functional abnormalities—specifically functional responses and reductions in functional connectivity. Indeed, the current study provides hallmark evidence of a neurometabolic imbalance in neurotransmission related to the networks sub-serving executive control and the alerting of attention, functions which have been previously implicated in ASD pathogenesis [14]. Furthermore, the neurometabolic differences between the two regions (the ACC and the PCC) are related to the symptom severity within ASD, as can be appreciated in Figure 2.

In this sense, a plausible hypothesis is that the neurometabolic microenvironment may determine the modulation and trafficking of distinct connectivity configurations. Could the changes in specific metabolites, detected by ^1^H-MRS, predict who is at risk of developing ASD? To try to answer this question, we addressed a critical gap in the literature by examining the metabolites intrinsic to the brain in adults with ASD at the time of identifying the specific metabolic differences between the ACC and PCC (hubs of salience and the DMN) that could be most useful for discriminating adults with ASD from their TD peers. We used a well-established ^1^H-MRS model, a paradigm based on spectroscopy, which is a technique used mainly in the elucidation of molecular structures, although it can also be used for quantitative purposes and in kinetic and thermodynamic studies [38].

To explore this issue, we investigated herein the putative NAAG metabolism alterations in the relationships within specific brain areas, namely, the ACC and PCC, and their subsequent impact on ASD. Our findings demonstrated that deregulation of the NAAG and, consequently, of its entire metabolic pathway have profound effects on the neurochemical imbalance present in subjects with ASD. Furthermore, these changes in NAAG trafficking may be extended to other biomarkers related to neurodegenerative diseases, such as Parkinson’s disease (PD), Alzheimer’s disease (AD), and other disorders such as schizophrenia, amyotrophic lateral sclerosis, traumatic brain injury, and glutamatergic receptors.

Numerous reports over the last 30 years suggest a link between mitochondrial dysfunction and ASD, based on findings of lactic acidosis, elevated urine levels of Krebs cycle metabolites, plasma carnitine deficiency, and decreased brain glucose utilization and adenosine triphosphate (ATP) levels in autistic patients [39]. In follow-up work, a bioenergetic deficiency in children with ASD has been confirmed by detecting a variety of abnormal biomarkers in the brain, plasma, cerebral spinal fluid (CSF), urine, fibroblasts, skeletal muscle, and buccal mucosa [40,41,42], showing biochemical markers of abnormal aerobic respiration [42].

These findings were confirmed in yet another postmortem analysis [43], in which investigators reported the reduced protein expression of various subunits of complexes I, III, IV, and V in the motor cortex, thalamus, and cingulate gyrus of the autistic brain compared to controls [43].

From ^1^H-MRS, variable levels of metabolic markers have been described in different brain regions in adults with ASD [13,44]. However, the results of the ^1^H_MRS measurement of lactate have been less consistent and may be related to challenges in the use of this technique [44,45]. On the whole, the data from ^1^H-MRS suggest metabolic dysfunction in the autistic brain.

Herein, we presented evidence demonstrating this potential connection with mitochondrial dysfunction. We focused specifically on biochemical links, between ACC and PCC. We evaluated the different metabolic peaks in the ACC and PCC, obtained by ^1^H-MRS, in subjects with ASD compared to healthy subjects. The absolute metabolic concentrations account for additional factors such as tissue type and relaxation time, and it was necessary to apply a reference scan with water suppression acquired using LCModel software, which analyzes an observed in vivo spectrum from brain tissue as a linear combination of in vitro solution spectra. The default model spectrum provided by the LCModel was acquired from in vitro measurements of metabolic solutions using the same pulse sequence and scanning parameters as the in vivo measurement.

In the present study, a Cr peak was detected, which is an important indicator of energy production and ATP creation, and was used as an internal reference to calculate the concentration of the other metabolites in ^1^H-MRS. Furthermore, the absolute Cr concentration was increased in previous studies in the auditory cortex of adults with ASD [45], suggesting that overall cellular energy metabolism might be elevated in ASD. In line with this, it has also been shown in vitro that glial cells contain a 2–4-fold higher concentration of creatine than do neurons [30]. Meanwhile, myoinositol is primarily located in glial cells and is interpreted as a marker of gliosis and glial cell numbers. The increase in the proportions of NAA/Cr and NAA/mI found herein reflect that the glia–neuron junctions are higher and, consequently, that the neurons function more, evidencing a process of active gliosis in the subjects corresponding to the AQ2 threshold. In fact, the distinctive character of astrocytes could be an important factor for high-level neuronal processing of the human brain and could act in a novel way related to oligodendrocytes in ASD.

In this sense, the increase in the proportion of NAA/Cho in the PCC is associated with an increase in mitochondrial function, as demonstrated by [21], as well as a neuronal attenuation anomaly or metabolic and synaptic pruning, shown in adults with Asperger’s syndrome [15]. Although, the decrease in NAA and NAA/Cr levels in the ACC indicates both the reduction and degradation of neurons and axons, there was a relevant abnormal increase in NAA/Cho in the PCC, which has not been described previously in ASD. Note that the peak of Cho in the spectroscopy included both acetylcholine and phosphatidylcholine, allowing a greater turnover in the cell membrane, which explains our results.

The synthesis of acetylcholine, which occurs only in cholinergic neurons, is an important component of the cell membrane. This cholinergic pathway innervates all cortical areas that potentially influence aspects of behavior and cognition according to [46], disrupted in ASD. According to another work [34], the social–behavioral deficits in ASD are specifically linked to aberrant connectivity in the PCC, and it is plausible that altered connectivity of this system is caused by a neurochemical imbalance in the PCC, which may contribute to deficits in socials skill in ASD.

Regarding our study, Figure 3 illustrates the trajectories of the neurometabolic variety across the autism spectrum, from AQ1 to AQ4 (pertaining to autism severity), both in the ACC and in the PCC (individual trajectories are shown in Supplemental Figures S1–S8. Consequently, when calculating the proportions of the NAA/Cr, NAA/mI, and NAA/Cho neurometabolites, we found a disproportion that could add to the severity of autism, reflected in the cingulated cortices (see Figure 4). However, although there are metabolic abnormalities in the brain in autism, a meta-analysis of proton magnetic resonance spectroscopy studies [13] showed that it is unsuitable for us to consider these results, because all of the ^1^H-MRS studies had different population characteristics, evaluated distinct brain regions, and used different ^1^H-MRS parameters.

Herein, we showed differences in the NAA/Cho and NAA/mI ratios as a contribution to the pathogenesis of autism, revealing a characteristic metabolic pattern that can be discriminated by spectroscopy and may even be considered as novel phenotypic neuroplasticity in ASD, if considering the differences in the NAA/Cr ratio between AQ2 (with the average AQ test values of the typical population) and AQ4 (with a very high index of autistic characteristics) shown in the PCC. All of this is in opposition to the reports of patients with lateral medullary infarction LMI (a mild cognitive impairment, a term used to describe the pre-Alzheimer’s stage) that later developed into AD, and patients with probable AD [47] in the same brain region. In addition, if we consider the NAA/Cho ratio in AQ1 as our metabolic TD reference, the evidence suggests that subjects within AQ3 (above average autistic characteristics) are deficient and/or hypometabolic across the entire spectrum. It was taken into consideration that choline is usually a marker of cellular density, which would reflect the damage in cholinergic neurons. However, both choline and myoinositol are also linked to glial cells and their membrane, for which these metabolites have been specifically linked to aberrant connectivity in the PCC [33], but the mechanism is not fully clear.

Although the absolute metabolic concentration in the brain is an indirect measure of cellular properties and, therefore, we can only infer the atypical cellular potential in regions of interest, these neurochemical findings will help to better establish the timing and mechanisms underlying the genetic abnormalities known to be involved in at least some cases of autism [46]. Thus, we consider there to be a need for further research to elucidate this unknown factors.

Our study hypothesis, that patients with ASD would have different metabolic in the cingulated cortices, was supported by the results obtained: a significant increase in the Glu concentration, related to neurotoxicity [48,49,50], in the anterior cingulated cortex, as well as a significant decrease in the NAAG concentration in the posterior cingulated cortex, in subjects with ASD. In this sense, a significant increase in the concentration of NAAG detected in the AQ4 group revealed severe hypomyelination in the ACC, according to a very high index of autistic characteristics, in agreement with other studies where a high NAAG concentration was associated with severe hypomyelination in cases of hypomyelinating leukodystrophy [51]. This explains the low functional connectivity in the resting state of the DMN described in ASD [44].

In contrast, hyperglutamatergia in the ACC was involved in the pathophysiology of the AQ3 and AQ4 groups, where glutamate plays an important role in autism pathogenesis [49]. This phenomenon may directly influence their association with clinically meaningful behavioral differences, which may be widely used as an in vivo marker of neurometabolic fitness.

A hallmark in the current study was the imbalance of the NAA/NAAG ratio in AQ2, AQ3, and AQ4 with respect to AQ1, evidencing possible changes across the autism spectrum in the cingulated cortices. As we mentioned in the introduction, the ACC is the region that receives projections mainly from the amygdala and, together with the dorsolateral prefrontal cortex, intervenes in the regulation of behavior and plays an important role in changing attention during a working memory operation. Consequently, lesions in the ACC interfere with selective attention, monitoring of competitive responses, and self-initiation of behavior [12], which would relate to our neurometabolic findings.

In contrast to the ACC, the posterior cingulated cortex receives most of the projections from the hippocampus, forming the emotional–social component of the memory system [52,53]. Both the anterior and posterior cingulated cortices play a central role in both the ‘salience network’ and the ‘default network’, respectively, as demonstrated by the functional magnetic resonance imaging studies referenced above.

Likewise, the results of studies of the DMN have suggested that the PCC can be used as an area of interest, with success in both children and adults with ASD, because this area shows a robust activation in the state of rest (resting state); however, neurometabolically, little is known about the composition of this node [34,54,55]. Therefore, this study represents an important contribution to the knowledge of this brain region.

An extra point in the current study was the development of an enzyme kinetics model, based on the assumption that if the enzyme (NAAGS) is absent, the reaction rate (V) is proportional to the concentration of the substrates (NAA and Glu) and equal to the concentration of the product (NAAG). Considering that the NAAG (product) concentration in the ACC was reduced by more than 50% in the autistic subjects belonging to the AQ3 group, this finding was used as a base for the NAAG kinetics reaction study in the substrate inhibition model using the Michaelis–Menten equation, under the assumption that the concentration of the enzyme–substrates complex NAAGS(NAAGlu) remains constant. The development of the enzyme kinetics by substrate inhibition allowed us to calculate the catalytic efficiency of the enzyme (NAAGS) in NAAG biosynthesis, to compare the utilization of the different substrates (NAA and Glu), and to measure the substrates’ specificity (Km and Ki) or relative suitability when both reacting with the enzyme.

Furthermore, the theory of induced fit postulates that the binding of a substrate can induce a noticeable change in the orientation of reactive groups at the active site of an enzyme and that catalysis often occurs only when these reactive groups have a specific orientation. In this case, the enzyme reaction with two substrates (NAA and Glu) can exhibit random-ordered or compulsory-ordered reactions with the formation of a ternary complex.

The Glu concentration was significantly higher in the ASD (specifically AQ3 and AQ4) subjects in the ACC compared to the TD subjects, which led us to suppose an essential feature of the Michaelis–Menten kinetics, where the catalyst becomes saturated at high substrate concentrations. As a consequence, the reaction rate (V) does not increase indefinitely with the substrate concentration; But approaches a limit (V_max_), and when V = V_max_, all of the enzyme is bound to substrate in the form of an enzyme–substrate complex.

Regarding the statistical results of R^2^, the study developed here has an inherently greater amount of unexplained variation. Metabolic variations in the brain are more difficult to predict than things such as physical processes, so they are meant to have lower R^2^ values. Regression models with low R^2^ values can be perfectly good models for several reasons. It is important to mention that our main interest was to understand the relationships between the variables; therefore, a low R^2^ value does not deny the importance of any significant variable (e.g., glutamate and NAAG concentrations in the ACC). Even with a low R^2^ value^,^ statistically significant *p*-values continue to identify relationships and the coefficients have the same interpretation [56]. Therefore, we did not have any additional cause to discount these findings.

Related to kinetic reaction of *N*-Acetyl-aspartyl-glutamate metabolism, we showed herein a decrease in Km for the NAA and Glu substrates, both in the ACC and in the PCC in ASD subjects. However, this does not mean that the enzyme was not present, since these constants are independent of the concentration of enzymes and only depend on the K-Off/K-On rate constants for the union of the substrate–enzyme complex. Interestingly, our findings indicate that a small amount of substrate can increase the reaction rate both rapidly and linearly in the ACC, suggesting that the active sites of the enzyme are saturated with the substrate. Furthermore, the enzyme–substrate complex was very tight and rarely dissociated for the substrate when reacting to provide the product, showing that the affinity between substrates and enzymes is significantly higher in individuals with autism—that is to say, the concentration of substrate necessary to reach a maximum kinetic rate (NAAGS).

As observed in the ASD group, the substrate inhibition reaction showed a progressive decrease in activity due to the high concentration of glutamate in the ACC. This may indicate the existence of more than one binding site between the substrate and the enzyme. However, at high substrate concentrations, the second site of inhibition became occupied, thereby inhibiting the enzyme. In this enzymatic reaction, both the substrate and the product were able to inhibit enzymatic activity. It is worth noting the possibility that the inhibition occurred as a result of the product (NAAG), since this is often a regulatory characteristic in metabolism and can be a form of negative feedback.

The enzyme kinetics imbalance in *N*-Acetyl-aspartyl-glutamate biosynthesis in autism between the anterior and posterior cingulated cortices is a novel finding indicative of a chronic neuroinflammatory state in these regions. This deregulation of the NAAG and, consequently, of its entire metabolic pathway (the amino acid NAA, the excitatory–inhibitory neurotransmitter Glu, and the neuropeptide–neuromodulator NAAG, in addition to the enzymes) appear as a possible cause of the neurochemical imbalance present in ASD, which could be the origin of the hypofunction of the SAN and DMN, as well as the temporal, visual, and motor frontal networks reported by other authors, which would explain not only the functional but also the neurochemical differences between the ACC and the PCC in ASD. To the best of our knowledge, this is the first demonstration of an imbalance of *N*-Acetyl-aspartyl-glutamate metabolism related to the cingulated cortices in ASD. Although still little explored in ASD, NAAG alterations may also be a consequence of injury to neurons, oligodendrocytes, and astrocytes, a product of oxidative stress process caused by deregulation redox metabolism linked to the reduced form of glutathione (GSH) [57]. In agreement with this, previous studies have demonstrated that autism is associated with deficits in glutathione antioxidant defense in selective regions of the brain. This suggests that disturbances in brain glutathione homeostasis may contribute to oxidative stress, immune dysfunction, and apoptosis, particularly in the cerebellum and temporal lobe, and may lead to neurodevelopmental abnormalities in autism [58].

Likewise, this relates the high vulnerability to oxidative stress and the low capacity of the methylation reaction with the clinical symptoms in children with ASD [59], as well as mitochondrial dysfunction and neuroinflammation, which have been identified as determinants of the appearance of ASD [60]. Based on the previous reference, and given the findings reported in this study of a significant decrease in the concentration of NAAG in the ACC and a significant increase in the PCC in subjects with autism, we can suggest the dysregulation of the NAAG as the main cause of a neurochemical imbalance that would support the etiology of autism.

We are aware that the ^1^H-MRS technique has a variety of parameters (echo time (TE) and repetition time (TR)), as one of its disadvantages when confirming scientific results; however, it allowed us to study a very important area at a cognitive level (social–emotional) [1,5,6,8,9,10,11,12], namely, the posterior cingulate cortex. Our metabolic PCC study can be contrasted with functional magnetic resonance imaging fMRI studies, which show that the posterior cingulate cortex presents functional abnormalities [2,3,4,7] in people with ASD. Emphasizing the importance of our findings, a hitherto unknown metabolic pathway would be very beneficial to delve into, highlighting the importance of this work when relating neurochemistry with the main brain networks.

## 4. Materials and Methods

### 4.1. Population

#### 4.1.1. Sample Size and Strength

This study was undertaken in accordance with the ethical standards and the Declaration of Helsinki of 1964, revised in 2000, and was approved by our local ethical committee. This study was approved by the Research Ethics Comities and Animal Welfares (CEIBA) (registration number: CEIBA2013-0056) of University of La Laguna. A small sample of subjects with ASD (*n* = 27) and TD (*n* = 44) was chosen, since this was an exploratory study. Similar sample sizes have been used in identifying significant differences in glutamate levels in different brain regions in patients with ASD and in controls before.

#### 4.1.2. Sample Recruitment, Sample Characteristics, and Study Adherence

Young adults diagnosed with ASD were enrolled from the three care centers for people with ASD associations in Tenerife (i.e., APANATE, ASPERTEN, and ALDIS). Adults with a childhood diagnosis of ASD without changes during growth, which was corroborated by the Spanish version of the Autism-Spectrum Quotient (AQ) test (Baron-Cohen S, 2005). The participants in this study were adults diagnosed with ASD during childhood by the respective psychiatry and psychology service of the University Hospital of the Canary Islands (HUC), Spain, by applying the tests used to evaluate and measure early childhood development (WISC [61], MSCA [62], IQ [63], CPM Scale [64], and Autism Screening CHAT [65], ADOS [66], and ADI-R1 [67]) without any variation in diagnosis during growth according to medical history. ADI-R or ADOS were not independently diagnosed on our part, considering that there was a high degree of agreement between clinical and research diagnoses [68], thus avoiding excessive diagnosis; however, this lack of diagnosis can be seen as a limitation of the methodology.

The study procedures were approved by the Canary University Hospital (HUC) and Magnetic Resonance Service for Biomedical Research (SRMIB), IMETISA, and informed consent was obtained for all study participants. The study was explained in simple language to the subjects and verbal assent was obtained from higher functioning adults who were capable of understanding the study process. The subjects were assessed with ^1^H-MRS. The Spanish version of the AQ test [69] was administered to all participants, including young TD adults. A small number of adults who met the criteria for ASD (*n* = 5), because of epilepsy, and TD (*n* = 1), because of glioma without symptoms, were excluded from the analyses. Adults were included in the analysis if they (1) had a successful, high-quality ^1^H-MRS result and (2) were assessed for an ASD diagnosis using the AQ test score. A total of 71 adults (27 ASD adults and 44 age-, sex-, and AQ-matched healthy comparison subjects) took part in the study and successfully (100% success rate) met these criteria; thus, they were included in the analyses, yielding two outcome groups: (1) ASD (*n* = 27; 22 males and 5 females), aged 17–23 years (mean, 20.6 ± 0.71 years), and (2) TD (*n* = 44; 19 males and 25 females) healthy controls aged 19–24 years (mean, 23.2 ± 0.71 years). All 71 subjects were right-handed (Table 1). The diagnosis of autism was established by a psychiatrist and psychologist in every case, and was verified through medical history.

By virtue of the differences of the AQ test results, we conducted follow-up analyses to assess whether the subgroups (AQ1, AQ2, AQ3, and AQ4) of the ASD and TD subjects, defined on the basis of autism symptom severity, differed in absolute metabolic concentrations in their cerebrum. The participants were stratified into subgroups according to established, empirically derived categories of the AQ test scores by Baron-Cohen, where the subgroup algorithm, which combines the scores on the five AQ domains (social skills, attention switching, attention to detail, communication, and imagination) derived the cutoff threshold to yield reliable autism subgroups [32]. This approach is consistent with previous publications on this sample, providing a description of the participant characteristics [70]. AQ1 (0–10 points) = below average; AQ2 (11–21 points) = average values of the normal population (the female mean is 15 and the male mean is 17); AQ3 (22–31 points) = above average; AQ4 (32–50points) = very high index of autistic characteristics (Asperger’s syndrome or high functioning autism has an average score of 35). We applied this same AQ threshold to stratify all participant subjects into AQ scores, and to identify them with the threshold shown (AQ1: *n* = 17; AQ2: *n* = 26; AQ3: *n* = 9; AQ4: *n* =13).

### 4.2. Proton Magnetic Resonance Spectroscopy (^1^H-MRS) Acquisition

Proton magnetic resonance spectroscopy (^1^H-MRS) is a non-invasive imaging method that provides spectroscopic information in addition to the image that is generated by magnetic resonance imaging MRI alone. The spectroscopic information obtained in a ^1^H-MRS study can be used to infer further information about cellular activity (metabolic information).

The methodology used in the development of this study was ordered according to the importance of the studies carried out, which led us to the reported results. For the first, the consistency of ^1^H-MRS was confirmed by detecting the metabolites present in the brain. The drawbacks in the application of ^1^H-MRS in intact tissues ‘in vivo’ have been reported as an inherent limitation of spectroscopy is the narrow range (5 ppm) in the chemical shift of non-exchangeable protons, where the resonance of a large number of metabolites overlaps. Therefore, it was necessary to fine-tune the technique before proceeding to developing the paradigm contained in the methodology used to quantify the detectable metabolites.

The absolute concentration of the metabolites present in each voxel was calculated, in the spectra resolved in time, that is, 68 spectra for each stimulus observed (i.e., 272 spectra). The presentation of each stimulus lasted 145.52 s (582.08 s in total); TR = 1070 msg and NEX = 2. The quantification was performed with the LCModel program, and the Tukey’s multiple comparisons test was applied in the statistical analysis. Furthermore, to optimize the acquisition of the spectra, it was necessary to modify several parameters to decrease the noise and artifacts of the spectroscopic signal, the most relevant parameters being the TE and the TR, with the former being the most relevant; currently, the TE used ‘in vivo’ by most groups varies between 18 and 288 ms. In this regard, we talk about studies with a short or long TE, using most studies with short TEs (between 18 and 45 ms) and studies with long TEs (between 120 and 288 ms), adducing different arguments in favor and against each option. On the contrary, in short TEs, a greater number of resonances are visible because the signal of compounds with strong modulation can be lost at long TEs. Thus, a short TE is necessary for a better evaluation of some compounds such as myoinositol, glutamine, and glutamate. However, there is no unanimous decision on TE to be used in clinical diagnosis [71,72].

The spectra were obtained by varying both the TE and TR values, which allowed us to define which were the best times for the spectra obtained to show the highest number of metabolites, based on the reliability indicators or lower levels of Cramér–Rao (<20%). Due to the characteristics and limitations of the area of interest, a voxel with a volume of 2 × 2 × 2 cm^3^ (8 mL) was used to obtain the average of the signals, which allowed us to elucidate spectra with optimal signal/noise ratios. Likewise, the acquisition time of the spectra (TE = 23 ms; TR = 1070 ms) was necessary for a better assessment of some compounds such as myoinositol or glutamate, according to the interest of our study.

In this way, the paradigm that was used throughout the research trajectory was established, considering also in the post-processing the main peaks of interest and their corresponding metabolites processed by the LCModel, taking in hand only the metabolites with a Cramér–Rao level [73,74] less than 20% (blue color). Finally, the paradigm used in all of the spectroscopy studies was as follows: TR = 1070 ms, TE = 23 ms, longitudinal magnetization rotation angle (flip angle) = 90°, matrix size = 256 × 256, NEX = 2, R^1^ = 13%, R^2^ = 30% and TG = 135 in a 2 × 2 × 2 cm^3^ voxel (8 mL). The data of all of the studies carried out and their results are available in the database of our Neurochemistry and Neuroimaging Laboratory.

All subjects underwent an MRI and ^1^H-MRS using a 3T Signa-HD MR scanner (GE Healthcare, Waukesha, WI, USA). T2-weighted images were used for positioning the volumes of interest (VOIs). The single voxel acquisition used a spin-echo sequence recorded within the following parameters: TE = 23 ms, TR = 1070 ms, NEX = 2, flip angle = 90°, and 256 acquisitions with the point-resolved spectroscopy (PRESS) technique. During data acquisition, the same experienced neuroradiologist, blind to the clinical data, placed the voxels (2 × 2 × 2 cm^3^) at the ACC and PCC. The main metabolite resonances were limited to 2.02 ppm for NAA, 2.04 ppm for NAAG, 3.03 ppm for Cr, 3.20 ppm for Cho, 3.55 ppm for mI, and, 3.77 ppm for Glu. We used a TE of 23 ms, as it is known that myoinositol can be readily detected in a short TE using ^1^H-MRS spectra of the brain due to its high concentration of (4–8) mM [71]. Each voxel was positioned so as to exclude contamination of signal from the skull and subcutaneous fat. Morphological examination enabled us to exclude other pathologies, such as congenital abnormalities, lesions in cerebral palsy, tumors, and hydrocephalus.

Contradictory, the signal from the neuromodulator NAAG and that of the amino acid NAA are considered difficult to separate in spectroscopy [75]; however, some works, using the construction of the phase space of the particles developed in our research group, revealed that it is possible to separate them due to NAAG having four visible attraction regions in the phase diagram space, considering the resonating protons of the acetyl-CH3 groups (2.04 ppm). Therefore, it is easy to deduce that the short- and long-term variability of the resonance process of NAAG is greater than that of NAA (2.02 ppm) and Glu (2.10 ppm) [76]. The paradigm used to obtain the spectroscopy in our studies allowed us to detect this neuromodulator with a %SD of <20%. Moreover, it was previously shown that NAAG and NAA can be discriminated through appropriate application of the MEGA-PRESS method by in vivo MRS at 3 Tesla (3T) [76].

Although an intense peak at 2 ppm is generally assigned to NAA (which is responsible for the greater part of the signal), in this work, it was assumed to correspond to NAA and NAAG. However, any small *N*-acetyl molecules in the brain will contribute to the peak. Furthermore, it was possible to measure NAAG at short echo times, suppressing the signals of multiplets from strongly coupled spin systems near 2 ppm, thus minimizing the interfering signals for detecting the acetyl proton signal of NAAG. One possible weakness of our method is the reliance on accurate suppression of the NAAG signal in the NAA scan, and particularly the NAA signal in the NAAG scan (due to the higher concentration of NAA). However, small contributions from other *N*-Acetyl species (e.g., *N*-Acetyl-glutamate) could result in overestimation of the NAA and NAAG concentrations.

Glutamine and glutamate (i.e., Glx and Glu/Gln) create a series of signals that are grouped into two regions (2.1–2.5: multiplets; 3.6–3.8: triplets). Some works suggest that a detailed analysis of the region allows to determine the contribution of each of the two components, but most study the entire region together. Recent studies with animal models in vitro suggest the existence of two pools of Glu and Gln related to the neuronal and glial compartments. Finally, it should also be noted that various studies suggest considering Gln as a glial marker.

### 4.3. Post-Processing and Neurometabolic Quantification

The ^1^H-MRS data sets were collected using single voxel acquisition techniques, performed using the SAGE software platform and LCModel, with no interaction or subjective input [73]. We took into consideration that the quantification of the absolute concentrations of brain metabolites, expressed in millimoles per kilogram of wet weight, involves the correction of many factors, including the tissue composition of the voxel (relative amounts of cerebrospinal fluid and grey and white matter), the T1 and T2 relaxation times of the metabolites in the patient, the location of the voxels and their relationship with the electromagnetic properties of the coil, and any temporary variation in the scanner [77]. Nevertheless, the assessment of metabolic ratios is generally the fastest and the most frequently employed method for the analysis of clinical ^1^H-MRS. The seven major resonances in the spectra (NAA, Cho, Cr, mI, NAA + NAAG, Glx, and Glu) were curved-fitted and peak areas were obtained from two voxels (ACC and PCC) and were used to calculate the metabolic concentration ratios of NAA/Cr, NAA/Cho, NAA/mI, Cho/Cr, and mI/Cr.

The absolute concentrations of the metabolites present in each voxel were calculated, in the spectra resolved in time, that is, 68 spectra for each stimulus observed (i.e., 272 spectra). Furthermore, the relative proportions of the different metabolites were calculated using creatine as reference, following standard clinical practice, where metabolic rates are evaluated using Cr, which is considered the most stable metabolite, as an internal reference [78].

The relative proportions of NAA/Cr, Cho/Cr, mI/Cr, NAA/Cho, and NAA/mI—in a first attempt to observe metabolic variations in the cingulated cortices of people with ASD—were compared with the results obtained from the healthy subjects. The use of creatine as a reference is documented by other studies, but not that of myoinositol or choline, which we considered important references due to their functions at the cellular level, and which can be used as markers of membrane integrity and cellular energy in the tissues studied [79,80,81]. Furthermore, the ratios of a metabolite do not represent all of the possible differences if a single specific denominator is used (as is the case with creatine), assuming that the signals recorded in all subjects were obtained in the same scanner unit and using the same protocol [82,83,84]. The relative quantification by means of the ratios of the various resonances, with respect to a metabolite as a reference, has the advantage that, for example, if there is an alteration in the concentration of some of the components in the ACC and PCC, it will result in different ratios and, consequently, it will be possible to observe this alteration (see Figure 4).

### 4.4. Data Analysis

Figure 1 demonstrates that the volume of interest (VOI) was located in the ACC and PCC regions, where we collected data on metabolites from two subject groups, i.e., ASD and TD. The spectra for each location were investigated separately and averaged. The results were processed with LCModel 6.3 to obtain absolute concentration estimates for the following metabolites in each location in each subject: Cr, NAA, NAA + NAAG, NAAG, Cho, mI, Glx, and Glu¸ considering the use of ratios does not necessarily assume one metabolite to be constant [73]. Furthermore, the ratios account for all non-metabolite-specific differences, and therefore can be reasonably compared across all participants scanned at the same institution with the same protocol.

#### 4.4.1. Enzyme Kinetics of N-Acetyl-aspartyl-glutamate in the Cingulated Cortices in ASD: A ^1^H-MRS Model

Living systems depend on chemical reactions which, on their own, occur at extremely slow rates. Enzyme kinetics studies the speed of chemical reactions that are catalyzed by enzymes. It is through the study of the kinetics and chemical dynamics of an enzyme that we can explain the details of its catalytic mechanism, its role in metabolism, how its activity in the cell is controlled, and how its activity can be inhibited by drugs, or powered by other types of molecules. One of the important characteristics of enzymes is that they act as catalysts that reduce the needed activation energy, so these reactions proceed at rates that are useful to the cell and differ greatly in the time they take to perform a catalytic cycle, which begins with the binding of the substrate and ends with the release of the product and the formation of the enzyme in its initial state. On the other side, the theory of induced fit postulates that the binding of a substrate; can induce a noticeable change in the orientation of reactive groups at the active site of an enzyme, and that catalysis often occur only when these reactive groups have a specific orientation. The hypothesis that arose in this point of the study is that NAAG synthetase (NAAGS) involucrate in NAAG biosynthesis, which could be indirectly measured from the concentration of *N*-Acetyl-aspartyl-glutamate, NAA, and Glu using the enzyme kinetics model. In this case, the enzyme reaction with two substrates (NAA and Glu) can exhibit random- or compulsory-ordered reactions with the formation of a ternary complex. Considering that the enzymes stabilize the transition states of the reactions and, a reduction in the activation energy is required. Using the brain ^1^H-MRS of individuals with ASD and TD controls, we measured the concentrations of the detected metabolites Glu and NAA within NAAG biosynthesis in the ACC and PCC, using the enzyme kinetics model to study the kinetics of the NAAGS enzyme in vivo.

Our goal was to investigate the imbalance of NAAG metabolism (as the only chemical derived from NAA metabolism) in the ACC and PCC; in order to make the study complete, it was essential to incorporate the analysis of NAAG synthetase, studying its chemical structure and physiology in NAAG metabolism, in addition to the relationship of the variations in its concentration and its correlation with clinical ASD.

The biosynthesis of NAAG was demonstrated in neural tissue explants, astrocytes, and neuroblastoma cells; however, no in vitro enzyme assay could be established, preventing purification of the enzyme [85]. Initially, enzymes synthesizing NAAG were not characterized and only the distribution of NAAG synthetase (NAAGS) expression was comparable to that of glutamate carboxypeptidase II (GCP-II), as reported. However, the high expression levels of NAAGS restricted to the brain has been strongly suggested to permit the identification of the gene that encodes NAAG synthetase [86].

Furthermore, the NAAGS sequence shows significant similarity to alfa-l-glutamate ligases (RimK family). These RimK proteins add glutamate residues to the C-terminus of the ribosomal protein S6. Moreover, most residues of the ATP-grasp domain that have been shown to be involved in ATP binding in the d-alanine:d-alanine ligase from *Escherichia coli* are also conserved in NAAGS [87]. The pre- and postsynaptic important effects of NAAG include the reduction of the concentration and the activity of glutamate; for this reason, it becomes, together with fibrillar astrocytes, another mechanism to avoid its toxic effects. Apart of this, NAAG is also considered essential for the progress of the neuronal interaction called synaptic plasticity, because its metabolism allows it to be sustained and efficient; to achieve this, it is essential to reconcile the presence of glial cells [18,30].

We used the ^1^H-MRS of individuals with ASD and healthy controls to measure the concentrations of the metabolites Glu and NAA as substrates involucrated NAAG biosynthesis in the ACC and PCC, with the intention to elucidate the kinetics of the NAAGS enzyme in vivo. Another interesting point is that both NAA and NAAG are present in neurons at very high concentrations—approximately 20 and 1 mM, respectively—but only approximately 0.1% of their content/min is released and, as a result, turn over approximately every 14–16 h. As a consequence, this rate of release coupled with their rapid hydrolysis (99.9%), will be present within the neuronal cytosol at any given moment [86], easily measured during spectroscopy.

Another important brain tissue characteristic is that NAA is targeted toward oligodendrocytes, which are the only cell type that can make catabolic NAA, while NAAG consists of a non-excitotoxic form of the neurotransmitter glutamate, which is targeted toward astrocytes [18]. Forget not, the formation of adduct of NAA and glutamate (NAAGlu), from which NAAG is synthesized using the enzyme NAAG synthase, which is the only metabolic pathway available for NAA in neurons. Taking into account the above, the study of enzyme kinetics can help us understand the function and regulation of enzymes [88].

The consideration of this study of the enzymatic kinetics for understanding the biosynthesis of the NAAG reaction, raised under the hypothesis of the NAAG synthetase concentration (NAAGS) that is involucrate in NAAG synthesis, could be indirectly measured from the concentration of *N*-Acetylaspartyl-glutamate, NAA, and Glu. Considering enzymes stabilize the transition states of the reactions, a reduction in the activation energy is required (as an important parameter). The Michaelis–Menten equation is one of the models that describes the activity of simple enzymes as a function of the substrate concentration. On the contrary, an essential feature of Michaelis–Menten kinetics is that the catalyst becomes saturated at high substrate concentrations, as is the case with NAA and Glu in the brain. In this case, we considered that the enzyme reaction with the substrates (NAA and Glu) can exhibit random- or compulsory-ordered reactions with the formation of a ternary complex, and that the catalyst can become saturated at high substrate concentrations.

For the development of data processing, the Michaelis–Menten equation is the one of the simplest and best-known approaches to enzyme kinetics. It takes the form of an equation relating reaction velocity to substrate concentration for a system where a substrate [S] binds reversibly to an enzyme (E) to form an enzyme–substrate complex (ES), which then reacts irreversibly to generate a product (P) and to regenerate a free enzyme (E) [89]. This system can be represented schematically as

(2) E + S ↔ ES → E + P

The Michaelis–Menten equation for this system is

(3) v=Vmax [S]/(KM+ [S])

where Vmax represents the maximum speed reached by the system at the maximum (saturation) concentrations of the substrate; the Michaelis–Menten constant (KM) is the substrate concentration at which the reaction rate is 50% of the V_max_; [S] is the concentration of the substrate.

Notwithstanding, another enzyme kinetics equations applied for development of our study was substrate inhibition. This equation is characterized by some substrates that also inhibit enzyme activity at high concentrations. This happens when two molecules of the substrate bind to the enzyme and thus block activity.

The equation model applied was

(4) $$Y=\mathrm{Vmax}*X/\left(\mathrm{KM}+X*\left(1+\frac{X}{\mathrm{Ki}}\right)\right)$$

where Vmax is the maximum enzyme velocity if the substrate did not also inhibit enzyme activity, expressed in the same units as Y; the Michaelis–Menten constant (KM) is expressed in the same units as X; Ki is the dissociation constant for substrate binding in such a way that two substrates can bind to an enzyme and is expressed in the same units as X. Rather than fit the enzyme progress curve, most analyses of enzyme kinetics (including all those built in to GraphPad Prism 8) can measure a product at a single time point. We consider each spectra of the resonance as a time point in which the concentrations of NAA, Glu, and NAAG corresponded kinetically in time.

The enzyme kinetics reaction which identifies the dipeptide synthetase, NAAGS (for NAAG synthetase), highly expressed in the nervous system can be represented schematically as

(5) $$\mathrm{NAA}\;+\;\mathrm{Glu}\;\begin{array}{c} \overset{\mathrm{NAAGs}}{\Leftrightarrow }\\ \mathrm{part}\;1 \end{array}\;\mathrm{NAAGs}\left(\mathrm{NAAGlu}\right)\;\underset{\mathrm{part}\;2}{\to }\;\mathrm{NAAGs}\;+\;\mathrm{NAAG}$$

We applied the substrate inhibition equation based on some simplified assumptions, part 1 of the equilibrium reaction, part 2 of the irreversible reaction, and E(NAAGs) and ES(NAAGsNAAGlu) (as the only forms of the enzyme). Only when these assumptions were met did Km correspond to the dissociation constant of the ES complex and Ki to the dissociation constants of part 2 of the reaction.

#### 4.4.2. Statistical Analysis

Numerical data were represented either as the mean ± SEM or normalized to young, healthy controls (the TD group), based on clarity. The statistical significance and the statistical analysis performed are detailed in every figure, as calculated using GraphPad Prisma v 8.0 (GraphPad Software, Inc. La Jolla, San Diego, CA, USA). The measure of absolute concentrations by ^1^H-MRS, in the anterior and posterior cingulated cortices of subjects with ASD and healthy controls, were considered when the approximate percentage of the maximum likelihood estimates of the metabolic concentrations and their uncertainties (Cramér–Rao of <20%) was not above 20% [72]; for the post-processing of the spectra, statistical analysis was carried out using the Mann–Whitney test (non-parametric) to evaluate the normal distribution of the absolute concentrations and ratios of the metabolites. Briefly, the intergroup differences were also compared, both in the absolute concentrations, as well as in the relative proportions of interest, using analysis of variance (ANOVA) for independent samples (one-way) in the two brain areas under study. To reduce the probability of finding significant alterations due to chance in each region, we used the Bonferroni correction for multiple comparisons in order to eliminate false positives once the analyses had been carried out, thus increasing the statistical power of the results. Differences of *p* < 0.05 were considered statistically significant. For comparisons (ASD vs. TD) the Student’s *t*-test was used to verify the differences in the relative proportions (metabolite x/metabolite y) studied at TE = 23 ms due to the non-normal distribution [56]. Group size also varied, which is indicated in the figure legends.

## 5. Conclusions

The main finding of this study was a significant decrease in the concentration of NAAG in the ACC, contrary to the PCC, where the concentration of this neuromodulator presented a significant increase. The deregulation of NAAG and, consequently, of its entire metabolic pathway (that is, all that it entails, since the amino acid NAA, the excitatory–inhibitory neurotransmitter Glu, and the neuropeptide–neuromodulator NAAG are involved, in addition to the NAAGS enzymes), looks like a possible cause of the neurochemical imbalance present in ASD, which could be the origin of the anatomical imbalance between cortical networks reported by Watanabe and Rees in 2016 [90]; this confirms the functional and neurochemical differences between the ACC and PCC in ASD.

We further conclude that a better understanding of the enzymatic activity in the synthesis of NAAG can lead us to a new therapeutic pathway in the treatment of individuals with ASD. Overall, this neuropeptide, in addition to being the cause of the hypoconnectivity between the main networks, is a very attractive pharmacological target for future investigations.

Finally, the determination of the concentration of NAAG creates a new and promising approach for intensified research on the glutamatergic systems and the effects of novel drug candidates on ASD.

## Figures and Tables

**Table 1:** Demographic characteristics of the data groups (*p* < 0.05). ASD, autism spectrum disorder; TD, typical development.

Characteristics	ASD (*n* = 22)	TD (*n* = 43)	*p* Value
Gender (male/female)	16/3	16/25	*p* = 0.016
Age(years)	20.58 (0.71)	23.19 (0.71)	*p* = 0.049
Familial hypothyroidism	15		*p* < 0.0001
Epilepsy	5		*p* < 0.0015
Gastointestinal disorders	17		*p* < 0.0001
Muscular hypotonia	14		*p* < 0.0001
Autism Quotient (0–50) points	33.84 (6.36)	11.67 (7.07)	*p* < 0.0001
Social skills (0–10) points	5.92 (2.54)	1.22 (1.61)	*p* = 0.039
Attention switching (0–10) points	6.81 (1.41)	3.49 (2.06)	*p* = 0.114
Attention to detail (0–10) points	4.3 (1.91)	5.03 (2.30)	*p* = 0.317
Communication (0–10) points	7.5 (1.75)	2.15 (1.63)	*p* = 0.942
Imagination (0–10) points	5.9 (1.82)	2.22 (1.59)	*p* = 0.542
Special education/transition to adulthood	6	0	*p* = 0.0008
Primary education	13	41	*p* = 0.0002
Secondary education	6	41	*p* = 0.0008
College	3	41	*p* = 0.0083

**Table 2:** Case–control analysis of absolute metabolic concentrations according to the cerebral regions: ASD vs. TD values for *N*-acetyl-aspartate (NAA), *N*-acetylaspartyl-glutamate (NAAG), glutamate + glutamine (Glx), glutamate (Glu), creatine (Cr), choline (Cho), and myoinositol (mI) (absolute concentrations are group means). Regions = anterior cingulate cortex and posterior cingulate cortex. *p* < 0.05 considered significantly different, while n.s. represents non-significant results.

Brain Region [mM]	ASD (*n* = 22)	TD (*n* = 43)	*p* Value
**Anterior Cingulated Cortex (ACC)**			
NAA + NAAG	9.78 (0.49)	10.44 (0.29)	*p* = 0.02
NAA	9.37 (1.36)	9.91 (0.68)	n.s.
NAAG	0.41 (0.27)	0.55 (0.13)	*p* = 0.02
Glx	16.10 (6.87)	15.19 (9.02)	n.s.
Glu	12.10 (3.92)	10.54 (5.64)	*p* = 0.02
Cho	2.08 (0.14)	2.08 (0.13)	n.s.
Cr	6.98 (1.56)	7.40 (1.87)	n.s.
mI	5.40 (0.78)	5.25 (0.27)	n.s.
**Posterior Cingulated Cortex (PCC)**			
NAA + NAAG	10.80 (0.86)	11.02 (0.68)	n.s.
NAA	10.47 (1.39)	10.68 (0.20)	n.s.
NAAG	0.34 (0.53)	0.32 (1.38)	n.s.
Glx	13.87 (4.09)	14.08 (2.15)	n.s.
Glu	10.22 (3.19)	10.71 (2.06)	n.s.
Cho	1.55 (0.44)	1.61 (0.38)	n.s.
Cr	6.72 (0.90)	6.99 (0.42)	n.s.
mI	4.98 (0.68)	5.13 (1.94)	n.s.

**Table 3:** Comparing the metabolite ratios between the ASD (n = 22) and TD (*n* = 43) groups in the ACC and the PCC using the Student’s *t*-test. m = average of the ratios, * = significant differences. *p* < 0.05 considered significantly different.

Ratios	NAA/Cr		mI/Cr		Cho/Cr		NAA/mI		NAA/Cho	
**ACC**										
ASD	1.64	0.02 *	1.09	0.04 *	0.36	0.08	1.65	0.47	5.11	0.42
TD	2.33		1.53		0.45		1.64		5.31	
**PCC**										
ASD	2.26	0.08	1.17	0.38	0.31	0.43	2.02	0.13	7.25	0.01 *
TD	1.90		1.13		0.32		1.76		5.96	

**Table 4:** Comparing the NAA/Cr, NAA/mI, and NAA/Cho ratios within the ASD (*n* = 22) and TD (*n* = 43) groups between the ACC and the PCC. *p* < 0.05 considered significantly different.

Ratios	NAA/Cr	*p* Value	NAA/mI	*p* Value	NAA/Cho	*p* Value
**ASD**						
ACC	1.37	n.s	2.06	n.s	5.07	0.0001
PCC	1.56		2.15		6.95	
**TD**						
ACC	1.59	n.s	2.03	n.s	5.02	<0.0001
PCC	1.52		2.18		6.85	

**Table 5:** Absolute metabolic concentrations in the applied Autism-Spectrum Quotient (AQ) threshold (AQ1 = 28.3%, *n* = 17; AQ2 = 43.3%, *n* = 26; AQ3 = 10.0%, *n* = 9; AQ4 = 18.3%, *n* = 13) to stratify the adults in the ASD and TD groups together. *p* < 0.05 considered significantly different.

Metabolite [mM] ± SD	AQ1 (0–10)	AQ2 (11–22)	AQ3 (23–31)	AQ4 (32–50)
**ACC**				
Cr + PCr	7.48 ± 0.81	7.42 ± 1.86	6.53 ± 0.98	7.72 ± 1.77
mI	6.12 ± 0.81	5.47 ± 1.42	7.29 ± 3.61	4.83 ± 1.93
GPC + PCho	1.83 ± 0.49	2.26 ± 0.59	1.63 ± 0.70	2.18 ± 0.63
NAA + NAAG	10.41 ± 0.79	10.66 ± 2.73	8.46 ± 0.49	10.56 ± 2.57
Glu + Gln	13.74 ± 4.06	15.67 ± 4.13	17.34 ± 1.43	16.65 ± 7.24
NAAG	0.43 ± 1.06	0.56 ± 0.97	0.21 ± 0.46	0.81 ± 1.39
Glu	8.04 ± 1.62	10.25 ± 2.70	13.87 ± 2.13	13.14 ± 4.14
NAA	9.96 ± 1.27	9.95 ± 2.38	8.41 ± 0.38	9.71 ± 1.78
**PCC**				
Cr + PCr	7.05 ± 0.45	6.69 ± 1.38	6.21 ± 0.69	6.68 ± 0.51
mI	5.99 ± 1.79	4.80 ± 0.71	5.51 ± 0.99	4.91 ± 0.83
GPC + PCho	1.63 ± 0.39	1.66 ± 0.41	1.29 ± 0.36	1.48 ± 0.32
NAA + NAAG	10.28 ± 2.60	10.79 ± 2.08	9.60 ± 0.95	10.98 ± 1.24
Glu + Gln	13.37 ± 2.11	13.76 ± 0.83	10.39 ± 0.37	14.14 ± 5.45
NAAG	0.35 ± 1.38	0.35 ± 0.51	0.41 ± 0.45	0.30 ± 0.38
Glu	10.51 ± 2.48	9.74 ± 2.15	8.94 ± 2.66	10.10 ± 2.20
NAA	9.89 ± 2.16	10.47 ± 2.04	8.98 ± 1.21	10.57 ± 1.56

Note: creatine + phosphocreatine (Cr + PCr); myoinositol (mI); glycerophosphocholine + phosphocholine (GPC + PCho); *N*-Acetyl-aspartate + *N*-Acetyl aspartyl glutamate (NAA + NAAG); Glutamate + glutamine (Glu + Gln) or Glx.

**Table 6:** Metabolite ratios in the applied AQ threshold (AQ1 = 28.3%, *n* = 17; AQ2 = 43.3%, *n* = 26; AQ3 = 10.0%, *n* = 9; AQ4 = 18.3%, *n* = 13) to stratify the adults in the ASD and TD groups together. *p* < 0.05 considered significantly different.

AQ	NAA/Cr	*p*	NAA/mI	*p*	NAA/Cho	*p*
**AQ1**						
ACC	1.41		1.99		4.93	
PCC	1.55	0.10	2.08	0.25	6.90	0.0004
**AQ2**						
ACC	1.33		1.86		4.77	
PCC	1.52	0.0002	2.18	0.0003	6.47	<0.0001
**AQ3**						
ACC	1.40		1.64		6.20	
PCC	1.48	0.29	1.99	0.69	7.48	0.05
**AQ4**						
ACC	1.43		2.33	0.68	4.49	
PCC	1.58	0.05	2.24		7.00	<0.0001

**Table 7:** Differences in the NAA/NAAG ratios in AQ2, AQ3, and AQ4 with respect to AQ1, evidencing changes in all autism spectrum groups. *p* < 0.05 = significant.

Index AQ	NAA/NAAG Ratio ACC	t	NAA/NAAG Ratio PCC	t
AQ1	28.77	-	28.77	-
AQ2	17.38	0.70	30.43	0.70
AQ3	42.90	1.64	24.46	0.32
AQ4	12.46	0.03	36.13	0.49

**Table 8:** Parameters for the expression of the biosynthesis of *N*-acetylaspartyl-glutamate in the substrate inhibition model of Michaelis–Menten by proton magnetic resonance spectroscopy (^1^H-MRS) in the ASD (*n* = 22) and TD (*n* = 43) groups.

Brain Region	V_max_ [μM/min]	ASD K_M_ [mM]	Ki [mM]	R^2^	V_max_ [μM/min]	TD K_M_ [mM]	Ki [mM]	R^2^
**ACC**								
**NAA**	1.1 × 10^8^	5.80 × 10^6^	5.59 × 10^−8^	−27.05	2.8 × 10^8^	3.2 × 10^7^	3.0 × 10^−8^	−86.74
**Glu**	1.6 × 10^8^	5.92 × 10^6^	4.44 × 10^−8^	−9.76	3.0 × 10^8^	5.1 × 10^7^	6.0 × 10^−8^	−16.96
**PCC**								
**NAA**	16.27	2.92 × 10^−3^	1.35	−23.95	---	---	---	---
**Glu**	19.13	4.68 × 10^−3^	1.04	−14.72	11.29	4.73 × 10^−4^	15.26	−20.33

## Data Availability

The raw data supporting the conclusions of this manuscript are present in the spectroscopy sample data.

## Supplementary Materials

The following are available online, Figure S1: Individual trajectories of (Glu + Gln), Figure S2: Individual trajectories of (Glu), Figure S3: Individual trajectories of (NAA), Figure S4: Individual trajectories of (Cr + PCr), Figure S5: Individual trajectories of (mI), Figure S6: Individual trajectories of (NAA + NAAG), Figure S7: Individual trajectories of (NAAG), Figure S8: Individual trajectories of (GPc + PCh).

## Sample Availability

Samples of spectroscopy resonance magnetic are available from the authors.
