# Supplementary material for: N-Acetylaspartyl-Glutamate Metabolism in the Cingulated Cortices as a Biomarker of the Etiology in ASD: A 1H-MRS Model
Source: Molecules. 2021 Jan 28;26(3):675. doi: 10.3390/molecules26030675 (PMC7866086; doi:10.3390/molecules26030675)
Supplement: Supplementary file 1 [file molecules-26-00675-s001.pdf]

Supplementary Materials

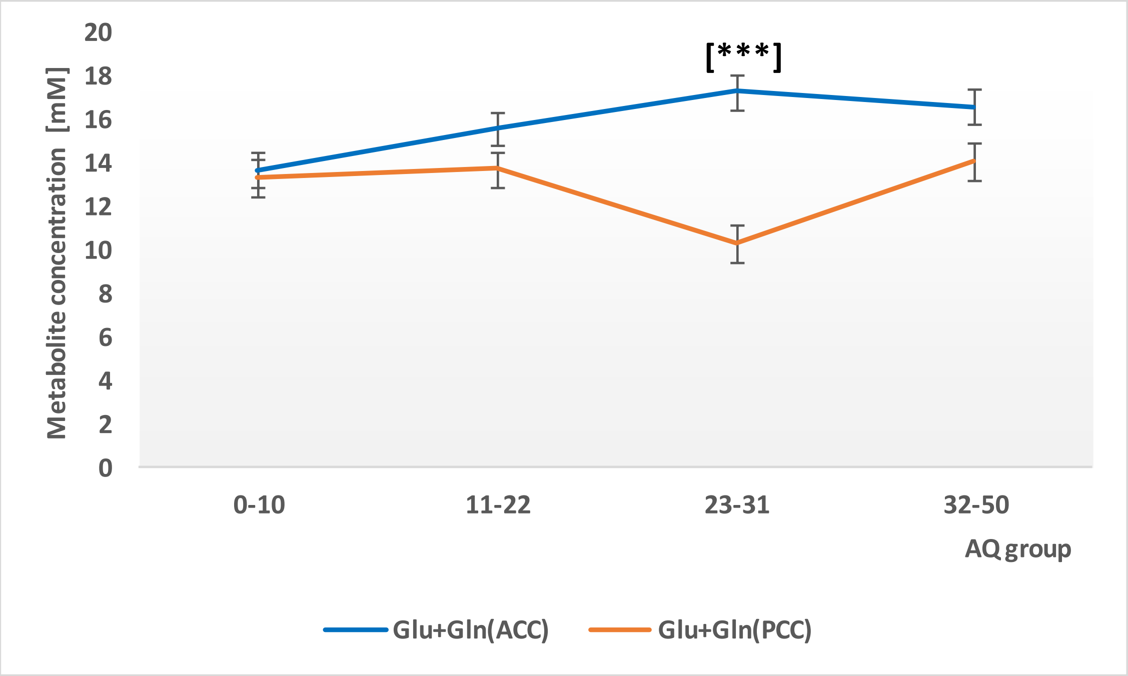

Figure S1. I Individual trajectories of (Glu + Gln); absolute metabolite concentration between ACC and PCC within the AQ test score. AQ1 = 28.3% (n = 17); AQ2 = 43.3% (n = 26); AQ3 = 10.0% (n = 9); and AQ4 = 18.3% (n = 13). Figure shows all subjects used for quantification. Graphs showing the quantification are included in Figure 2. $p < 0.05$ considered significantly different, [***] significant at $p < 0.001$ respectively.

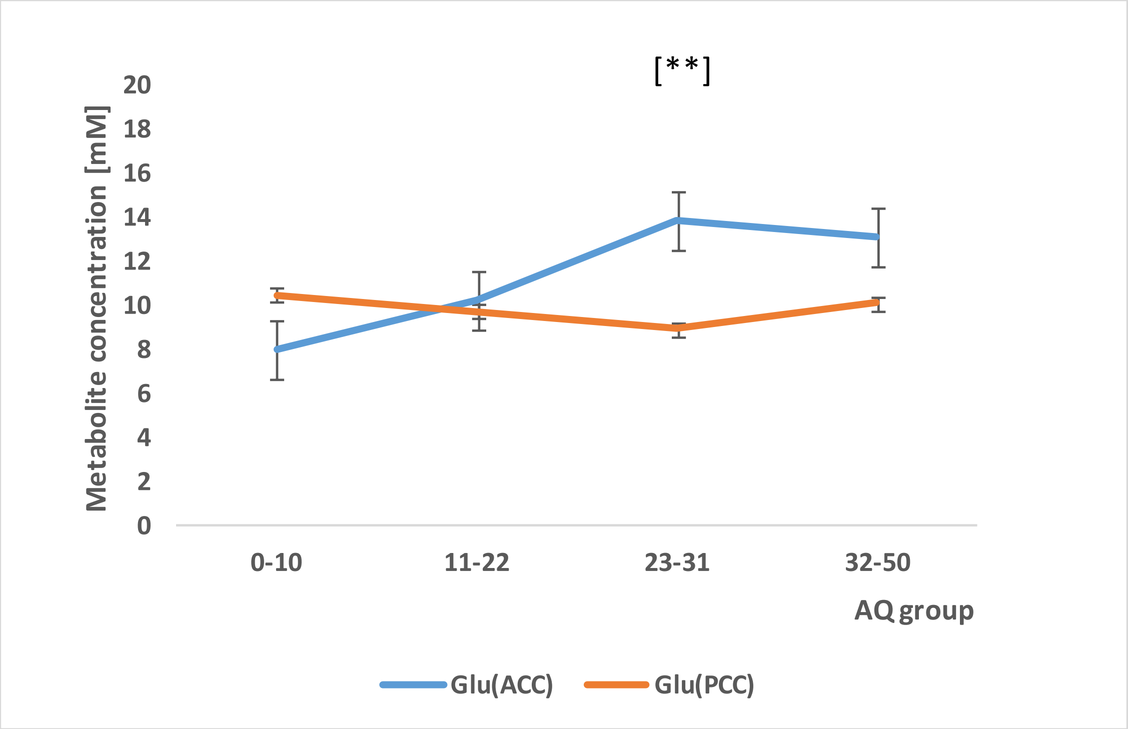

Figure S2. I Individual trajectories of (Glu); absolute metabolite concentration between ACC and PCC within the AQ test score. AQ1 = 28.3% (n = 17); AQ2 = 43.3% (n = 26); AQ3 = 10.0% (n = 9); and AQ4 = 18.3% (n = 13). Figure shows all subjects used for quantification. Graphs showing the quantification are included in Figure 2. $p < 0.05$ considered significantly different, [**] significant at $p < 0.01$ respectively.

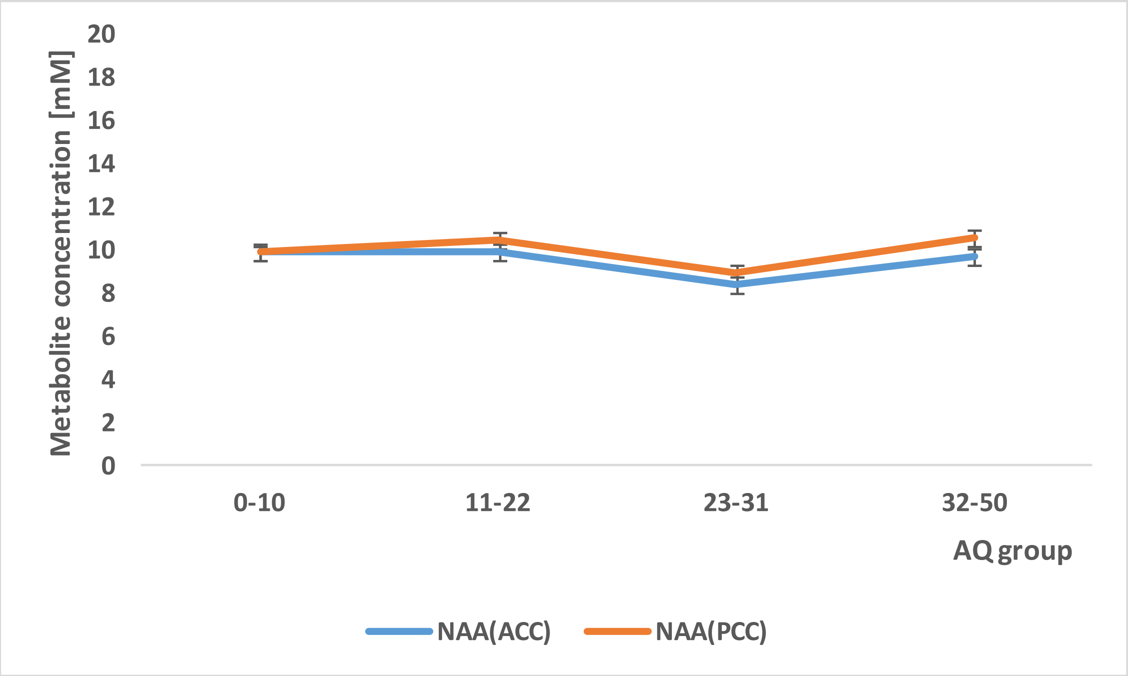

Figure S3. I Individual trajectories of (NAA); absolute metabolite concentration between ACC and PCC within the AQ test score. AQ1 = 28.3% (*n* = 17); AQ2 = 43.3% (*n* = 26); AQ3 = 10.0% (*n* = 9); and AQ4 = 18.3% (*n* = 13). Figure shows all subjects used for quantification. Graphs showing the quantification are included in Figure 2. $p < 0.05$ considered significantly different.

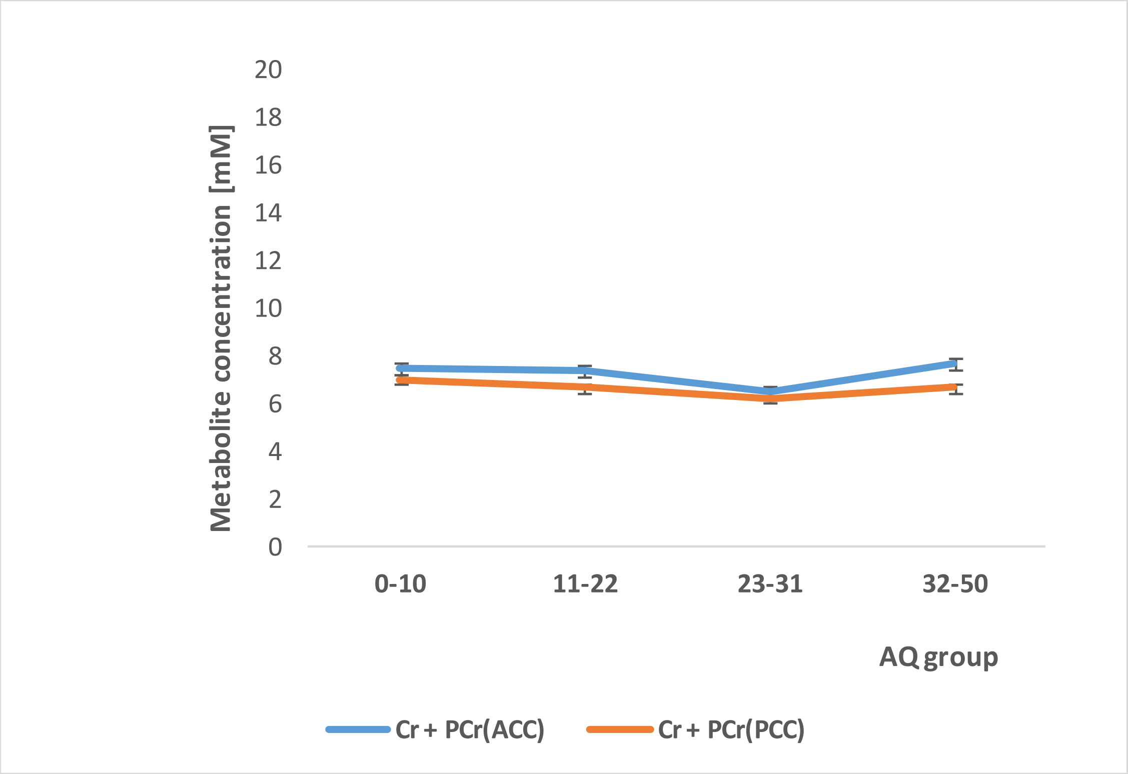

Figure S4. I Individual trajectories of (Cr + PCr); absolute metabolite concentration between ACC and PCC within the AQ test score. AQ1 = 28.3% (n = 17); AQ2 = 43.3% (n = 26); AQ3 = 10.0% (n = 9); and AQ4 = 18.3% (n = 13). Figure shows all subjects used for quantification. Graphs showing the quantification are included in Figure 2. $p < 0.05$ considered significantly different.

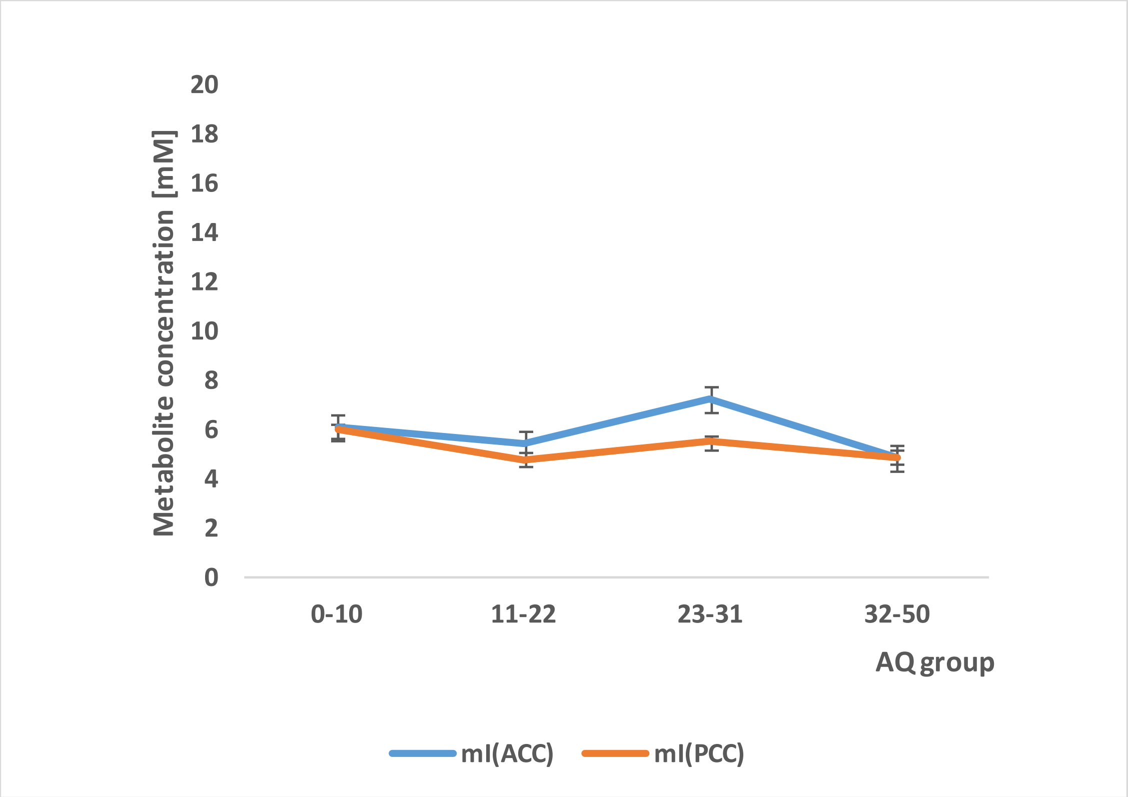

Figure S5. I Individual trajectories of (mI); absolute metabolite concentration between ACC and PCC within the AQ test score. AQ1 = 28.3% (n = 17); AQ2 = 43.3% (n = 26); AQ3 = 10.0% (n = 9); and AQ4 = 18.3% (n = 13). Figure shows all subjects used for quantification. Graphs showing the quantification are included in Figure 2. $p < 0.05$ considered significantly different.

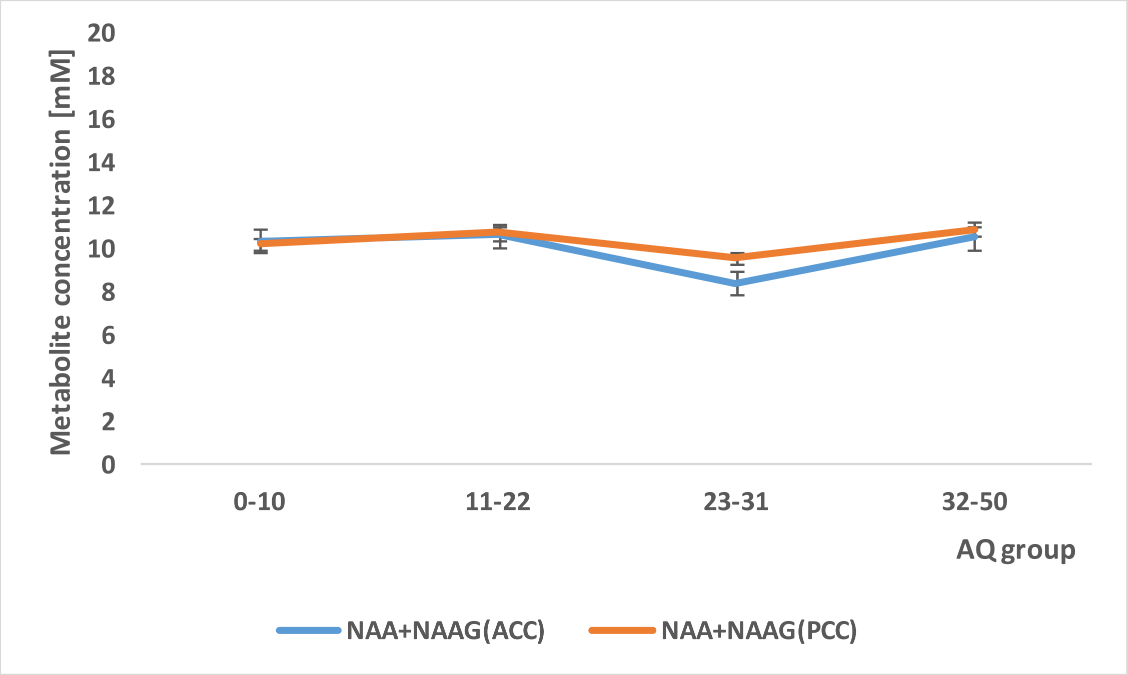

Figure S6. I Individual trajectories of (NAA + NAAG); absolute metabolite concentration between ACC and PCC within the AQ test score. AQ1 = 28.3% (*n* = 17); AQ2 = 43.3% (*n* = 26); AQ3 = 10.0% (*n* = 9); and AQ4 = 18.3% (*n* = 13). Figure shows all subjects used for quantification. Graphs showing the quantification are included in Figure 2. $p < 0.05$ considered significantly different.

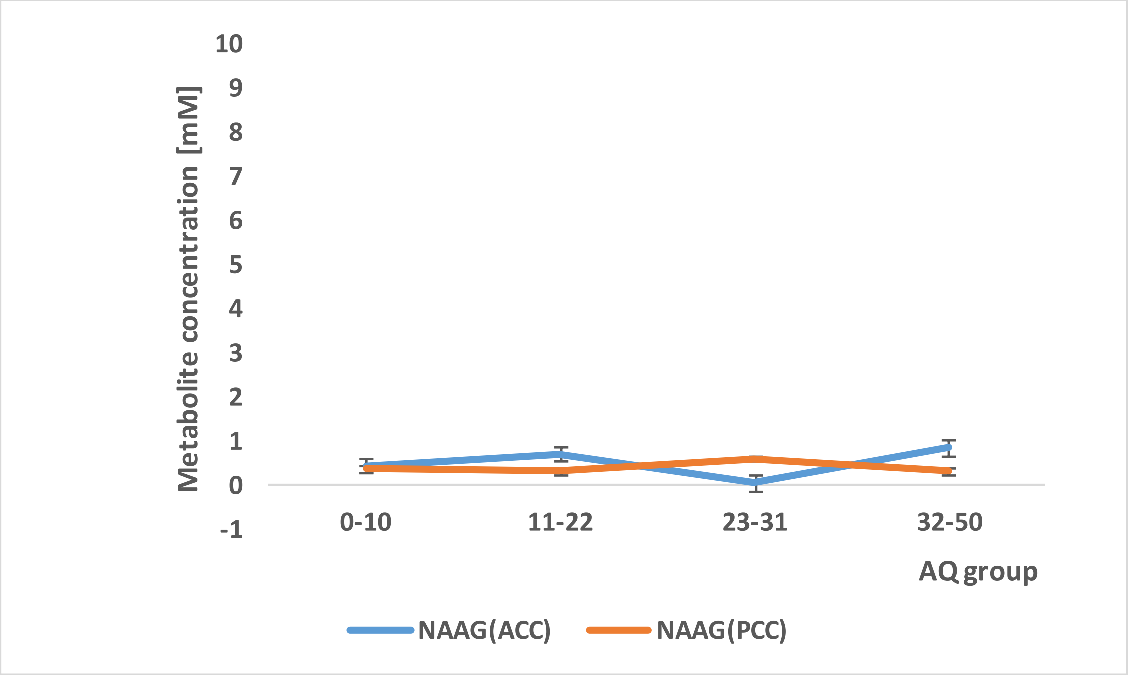

Figure S7. I Individual trajectories of (NAAG); absolute metabolite concentration between ACC and PCC within the AQ test score. AQ1 = 28.3% (n = 17); AQ2 = 43.3% (n = 26); AQ3 = 10.0% (n = 9); and AQ4 = 18.3% (n = 13). Figure shows all subjects used for quantification. Graphs showing the quantification are included in Figure 2. $p < 0.05$ considered significantly different.

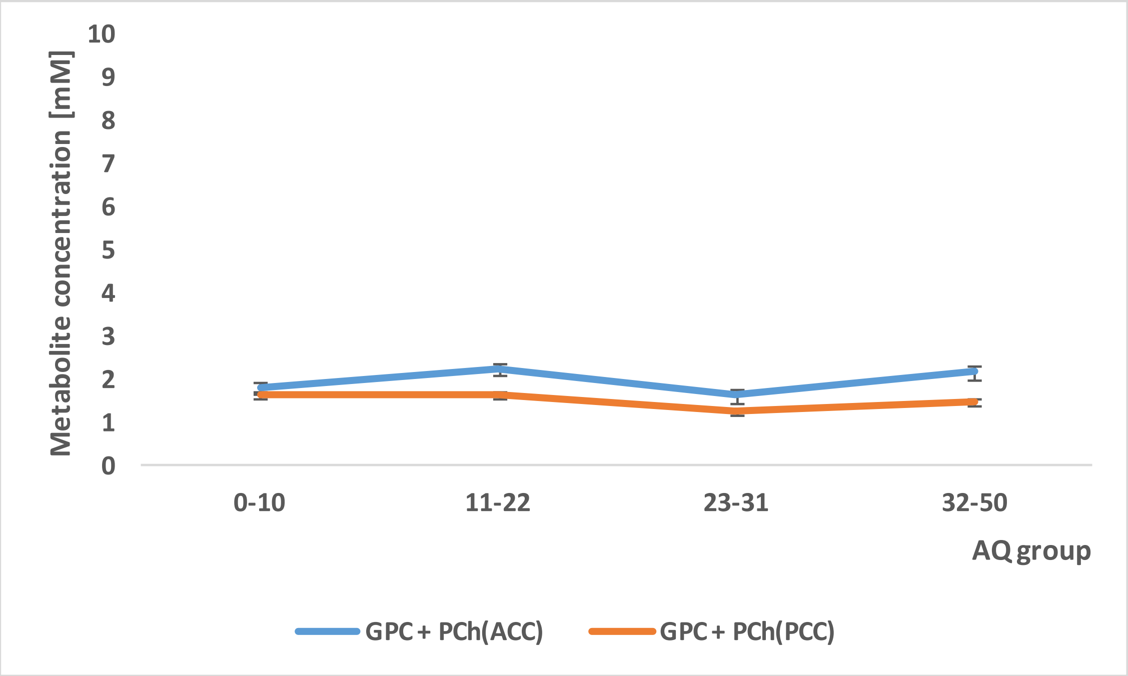

Figure S8. I Individual trajectories of (GPc + PCh); absolute metabolite concentration between ACC and PCC within the AQ test score. AQ1 = 28.3% (n = 17); AQ2 = 43.3% (n = 26); AQ3 = 10.0% (n = 9); and AQ4 = 18.3% (n = 13). Figure shows all subjects used for quantification. Graphs showing the quantification are included in Figure 2. $p < 0.05$ considered significantly different.
